# Resveratrol regulates nickel oxide-induced kidney damage by targeting the PERK/ATF-6/IRE1 and Nrf2/HO1 pathways

**DOI:** 10.1007/s00210-026-05107-0

**Published:** 2026-02-18

**Authors:** Orkun Barsbek Olcekci, Caglar Adiguzel

**Affiliations:** 1 Department of Biology, Graduate School of Natural and Applied Sciences, Gazi University, 06500 Ankara, Turkey; 2 Department of Biology, Faculty of Science, Gazi University, Ankara, 06500 Turkey

**Keywords:** Nickel oxide, Resveratrol, Oxidative stress, Endoplasmic reticulum stress, Apoptosis, Histopathology

## Abstract

Nickel oxide (NiO) is one of the most toxic heavy metals and poses a danger to human and animal health by causing serious kidney damage. Resveratrol (RES) is a flavonoid with antioxidant and anti-inflammatory properties, abundant in grapes and fruits. The current study is a first to investigate how RES affects oxidative stress, apoptosis, endoplasmic reticulum (ER) stress, inflammation, and histopathology against NiO-induced nephrotoxicity in rats using molecular, histological, immunohistochemical, and biochemical methods. The study used 24 Sprague–Dawley rats, randomly distributed into 4 groups of 6 rats each. The groups were designated as control, RES (10 mg/kg), NiO (10 mg/kg), and RES plus NiO. For 28 days, the prescribed doses of NiO and RES were given to the rats through gavage. After receiving NiO for 28 days, rats showed elevated levels of blood urea, creatinine, and uric acid. It has led to a rise in malondialdehyde (MDA) levels, an indicator of oxidative stress, and a decline in the transcription factor nuclear factor erythroid 2–related factor 2 (Nrf2) and antioxidant enzymes (HO-1, SOD, CAT, GPx, and GST). NiO application also increased the levels of IL-1β, TNF-α, 8-OHdG, and caspase-3 and the expression of endoplasmic reticulum (ER) stress markers such as heat shock proteins (HSP70, HSP90), GRP78, PERK, ATF6, ATF4, IRE1, XBP1, and CHOP, leading to histopathological changes in kidney tissues. RES administration significantly improved histological appearance by reducing impaired renal biochemistry, increased oxidative stress, inflammation, and ER stress. These results demonstrated that RES reduced NiO-induced nephrotoxicity.

**Graphical Abstract:**

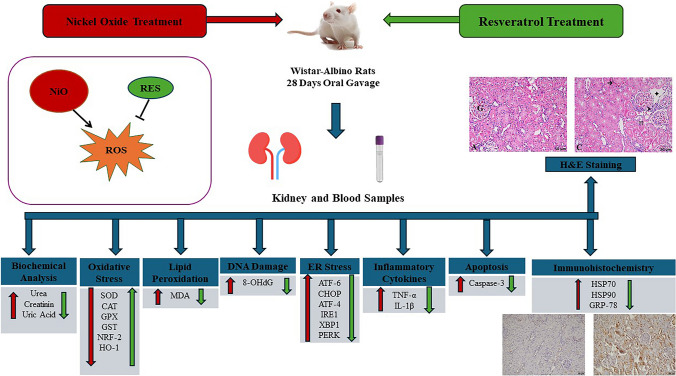

## Introduction

Nickel oxide is a chemical compound with a crystal lattice structure (Joyner et al. 2024). Due to its chemical structure, it shows high thermal stability, electrical conductivity, and catalytic activity, and as a result, it sees a wide range of use (Joyner et al. 2024). Ceramics, batteries, and alloys are the primary contenders in terms of nickel oxide usage, while electronic devices and sensors follow suit; nearly all of which are in frequent use and direct contact and exposure with humans (Manna and Bandyopadhyay 2019). Consequently, there has been much research on how nickel oxide might affect human health for both micro- and nanoparticles (Adiguzel et al. 2023; Jeong et al. 2022; Wang et al. 2023; Yang et al. 2024; Dumala et al. 2019; Noshy et al. 2022). Microparticles are defined as particles smaller than 0.1 μm, and nanoparticles are defined as particles smaller than 100 nm in size (Noshy et al. 2022; Wieland et al. 2022). This difference in size, along with the shape, surface-to-volume ratio, and chemical activity, results in different mechanisms for toxicity in human cells (Adiguzel and Karaboduk 2024). Microparticles can produce important results in terms of histopathological effects on tissues and organs (Lyons-Darden et al. 2023). Although NiO microparticles are highly important in terms of accumulation and toxicological effects, the lack of an in-depth toxicity mechanism in the literature made this study necessary.

Nickel oxide exposure can occur through the gastrointestinal, pulmonary, or dermal routes (Lyons-Darden et al. 2023). In cases of exposure, it mixes with the blood and accumulates in organs, for instance, the liver and kidneys (Dumala et al. 2019). Previous research has indicated that exposure to NiO has adverse effects on renal tissue and, as a result, pathological processes such as tubular necrosis, interstitial inflammation, and glomerular congestion occur (Dumala et al. 2019). In another study in which NiO was administered orally, intraperitoneally, and intravenously, decreases in antioxidant enzyme activities and increases in apoptotic marker expression were observed, as well as significant pathological findings in the histomorphology of rat kidneys (Karaboduk et al. 2024a).

It has been established that nickel oxide leads to oxidative stress, genotoxicity, and inflammation (Adiguzel et al. 2023; Jeong et al. 2022; Wang et al. 2023; Yang et al. 2024; Dumala et al. 2019; Noshy et al. 2022). Reactive oxygen species (ROS), likes of which are hydrogen peroxide (H_2_O_2_), hydroxyl radicals (•OH), superoxide anions (O_2_^−^), and singlet oxygens (O_2_), are highly reactive oxygen molecules which can be produced as a byproduct of metabolic activity, especially oxidative phosphorylation, and can even play a role in metabolism as a signaling molecule (Liu et al. 2023). When free radicals are present at excessive levels, they cause pathological damage by oxidizing DNA, lipids, and proteins (Liu et al. 2023).

Phenolics are secondary metabolites produced in response to stress in plants, with an emphasis on oxidative stress in plants (Duta-Bratu et al. 2023). Resveratrol is an antioxidant found mostly in grapes and fruits (Valletta et al. 2021; Duta-Bratu et al. 2023). It is a 14-carbon natural phenol with a C6–C2–C6 carbon structure, consisting of two benzene rings connected by an ethylene bridge, and it can exist in either cis or trans configuration (Valletta et al. 2021). It is known that it has anti-inflammatory, cardioprotective, and anticancer properties; increases the activity of antioxidant enzymes; and provides protection against lipid peroxidation (Diaz-Gerevini et al. 2016; Duta-Bratu et al. 2023). RES effectively cleans up hydroxyls, superoxides, and other radicals produced by metals thanks to its antioxidant properties. It acts as a metal chelator to protect cells from oxidative stress (Hafez et al. 2025). In a study where RES was used as an antioxidant, it was found that RES supplementation significantly improved liver toxicity and histopathological changes caused by silver particles administered to rats (Abdelrahman et al. 2022). In addition to its regulatory role in glucose and lipid metabolism, RES has healing properties on the cardiovascular system (Peng et al. 2025). RES is highly absorbed in humans when taken orally and is metabolized to sulfate conjugates in the liver and intestines and excreted in feces and urine (Yang et al. 2025a; Szymkowiak et al. 2025). The current study aims to investigate resveratrol’s potential protective function against possible damage of NiO microparticles on renal tissue with many biochemical, histopathological and molecular parameters.

## Materials and methods

### Reagents

Resveratrol was purchased from BLDpharm (CAS No. 501–36-0), and nickel oxide (44 µm, 95% purity) was purchased from Nanogarific Nano Technology (CAS No. 1313–99-1).

### Animals and experimental procedure

Animal experiments were conducted at Gazi University Laboratory Animal Breeding and Experimental Research Center. Twenty-four adult Sprague–Dawley rats (170–250 g) were obtained from this center. The animals were housed in special cages under standard laboratory conditions, with controlled temperature (20 ± 2 °C) and a 12-h light/12-h dark cycle. The rats had ad libitum access to standard food pellets and drinking water throughout the experiment. The animals to be used in the study are summarized in Table 1. Twenty-four animals were seperated into 4 groups, each group consisting of six (*n* = 6) animals, and administered via gavage once a day for 28 days.

**Table 1:** Design of experimental

Groups	Treatment
Control group	1 ml/kg bw corn oil
RES group	10 mg/kg bw per day in corn oil
NiO group	10 mg/kg bw per day in distilled water
RES + NiO group	10 mg/kg bw per day in corn oil RES + 10 mg/kg bw per day in distilled water NiO

In this study, the dose applied for NiO was selected based on the studies of Karaboduk et al. (2024a) and Singh et al. (2022a). The NiO dose to be applied for this study was determined as 10 mg/kg bw. Again, in this study, the resveratrol application dose was selected based on the study conducted by Ibrahim et al. (2021) in the past, which showed that it provided protection against environmental pollutants.

After the tested substances were applied for 28 days, the experimental animals were dissected under anesthesia with the combination of ketamine + xylazine. Biochemical analyses were performed with blood samples taken from their hearts, and molecular, histopathological, and immunohistochemical studies were performed with kidney tissues isolated after dissection.

### Body and organ weights

The weights of all animals in this study were recorded on the initial and final days of the 28-day application period, and after dissection, the kidney tissues were removed and weighed. The relative weights (%) of the kidney tissue were calculated as grams per 100 g of body weight.

### Bioaccumulation of nickel in the kidney

For nickel determination under the conditions recommended by the manufacturer, a Varian (Palo Alto, CA, USA) AA240FS flame atomic absorption spectrometer equipped with a deuterium lamp background corrector, a nickel hollow cathode lamp (Varian), and an atomizer using an air-acetylene flame was used. 0.3-g kidney tissue samples were digested in 3 ml nitric acid, mixed with H_2_O_2_, and then diluted to 10 ml with ultrapure water. The wavelength, lamp current, slit width, and acetylene flow rate for Ni were 232.0 nm, 4 mA, 0.2 nm, and 2 l min^−1^, respectively. Calibration standard solutions (1, 2, 4, and 8 mg/l^−1^) were prepared by diluting a nickel stock solution (Merck Darmstadt, Germany) with a concentration of 1000 mg/l^−1^. Calibration curves and equations were obtained by measuring the absorbance values of the element according to the concentrations of the working standard solutions (Yalçınkaya and Erdoğan 2012).

### Renal function tests

Serum urea, creatinine, and uric acid levels were analyzed for renal function. Urea (Otto Scientific, OttoBC157), uric acid (Otto Scientific, OttoBC158), and creatinine (Otto Scientific, OttoBC139) were analyzed colorometrically on the Mindray-BS400 automatic analyzer utilizing commercial kits.

### Analysis of oxidative stress parameters in renal tissues

For the analysis of oxidative stress parameters, renal tissues were homogenized with KCl and centrifuged at + 4 °C for 15 min. Protein levels in renal tissues were analyzed according to the method of Lowry et al. (1951). Malondialdehyde (MDA), superoxide dismutase (SOD), catalase (CAT), glutathione peroxidase (GPx), glutathione s-transferase (GST), nuclear factor erythroid 2 (Nrf2), heme oxygenase-1 (HO-1), 8-hydroxy-2’-deoxyguanosine (8-OHdG), and interleukin 1-Beta (IL-1β) were measured using the upper fluids of the homogenized tissues. The MDA level was measured by the method of Ohkawa et al. (1979), SOD activity by the method of Marklund and Marklund (1974), CAT activity by the method developed by Aebi (1984), GPx activity by the method of Paglia and Valentine (1987), and GST activity by the method developed by Habig et al. (1974). Nrf2 (BT-Lab, Cat No: E1083Ra), HO-1 (Elabscience, Cat No: E-EL-R0488), 8-OHdG (Elabscience, Cat No: E-EL-0028), and IL-1β levels (BT-Lab, Cat No: E0119Ra) were analyzed in a microplate reader by the ELISA method using commercial kits according to the manufacturer’s instructions.

### Quantitative real-time PCR analysis in renal tissues

The mRNA transcript levels of Protein Kinase RNA-Like ER Kinase (PERK), Activating Transcription Factor 6 (ATF-6), Activating Transcription Factor 4 (ATF-4), Inositol-Requiring Enzyme 1 (IRE1), C/EBP homologous protein (CHOP), and X-box binding protein 1 (XBP1) genes in kidney tissue were analyzed using the RT-PCR method. Primer sequences are presented in Table 2. Total RNAs were obtained from kidney tissue using a total RNA extraction kit (Invitrogen, Cat No: 15596026) and converted into cDNAs using the Otto synthesis kit (Otto Scientific, OttoP1152, Ankara). The obtained cDNAs were reacted with PERK, ATF-6, ATF-4, CHOP, XBP1, IRE1, and Actin-beta (Actb) as a housekeeping gene by the qRT-PCR method. The expressions of the relevant genes were measured by calculating the obtained data with the delta delta Ct (2-ΔΔCt) method (Livak and Schmittgen 2001).

**Table 2:** Primer sequences

Gene	Sequences (5ʹ−3ʹ)	Length (bp)	Accession nos
PERK	F: GCGGCAGGTCCTTAGTAATC R: ACCAGATCCCACGTCCAAAT	104	NM_031599.2
ATF-6	F: CCAAAGGTCAGACTGTTTTGC R: ACACAGTTTTCCGTTCACCG	179	NM_001107196.1
ATF-4	F: ACCATGGCGTATTAGAGGCAG R: GTTTCGTGAAGAGCGCCA	121	NM_024403.2
IRE1	F: TGGACGGACAGAATACACCA R: TGTAGTCCACGTCATCCTCG	113	NM_001191926.1
CHOP	F: TCTTCATACACCACCACACCTG R: AGGGATGCAGGGTCAAGAGT	228	NM_001109986.1
XBP1	F: CATTCTGGACAAGTTGGACC R: TGATGAGGTCCCCACTGACA	81	NM_001271731.1
β-Actin	F: ACAACCTTCTTGCAGCTCCTC R: CTGACCCATACCCACCATCAC	200	NM_031144.3

### Immunohistochemistry

HSP70 (Affinity, AF5466), HSP90 (Affinity, AF5368), GRP78 (Affinity, AF5366), TNF-α (Bioss, Bs-10802R), and caspase-3 (Bioss, Bs-2593R) were performed according to the procedures described by Adiguzel et al. (2025). Kidney sections of 5–6-μm thickness were passed through an increasing alcohol series. As a subsequent procedure, citrate buffer solution was applied for antigen retrieval. Sections were treated with 3% hydrogen peroxide for 15 min to mask endogenous peroxide. In the next step, 1/100 diluted antibodies were dropped onto the sections 15 min after UV serum block was dropped and kept at + 4 °C for 1 day. The secondary antibody HRP-streptavidin was then dropped onto the tissues and washed with PBS. Sections were then treated with DAB (3,3′-diaminobenzidine) chromogen and counterstained with Gill hematoxylin. To evaluate the immunoreactivity of HSP70, HSP90, GRP78, TNF-α, and caspase-3 antibodies, experimental groups were evaluated and their photographs were taken. A total of 100 measurements were taken from the experimental groups for each antibody. The measurements were analyzed in terms of immunoreactivity intensity with the Image J (1.53 k, National Institute of Health, USA) program. Then, statistical comparisons were made between the experimental groups.

### Histopathological analysis

Post-experimentation, renal samples were acquired and subjected to fixation in 10% neutral formalin. In the next stage, an increasing alcohol series, xylol, and paraffin processes were carried out. Five- to six-µm-thick sections were taken from the tissues that were turned into paraffin blocks with a microtome. A routine hematoxylin and eosin (H&E) staining procedure was applied, and permanent preparations were made. Photographs were taken from the prepared preparations using an Olympus BX-51 light microscope and an Olympus EP50 camera integrated into the microscope. In view of the morphological changes, the following criteria were used for histopathological evaluation. Inflammatory cell infiltration, hemorrhage, glomerular degeneration, tubular degeneration, and edema were observed in kidney tissue. The degree of renal damage was classified on a scale of 0–5 and scored as 0 = none, 1 = 0–20%, 2 = 21–40%, 3 = 41–60%, 4 = 61–80%, and 5 = 81–100% involvement (Akin et al. 2025). All sections in the study group were examined, and the quantitative data obtained were compared statistically.

### Statistical analyses

GraphPad Prism (GraphPad Software, La Jolla, California, USA) version 10 was used for statistical analyses. Shapiro–Wilk test was used to determine whether the data were normally distributed. For normally distributed data, one-way analysis of variance (ANOVA) and Tukey’s post hoc test were used for intergroup significance test. For non-normally distributed data, Kruskal–Wallis test and Dunn’s multiple comparison test were used to determine significance between groups. *p* < 0.05 was considered statistically significant.

## Results

### Body and organ weights

Table 3 shows the body and kidney weights of the rats. No significant difference in body weight was observed between the control group rats and the RES group rats (*p* < 0.05). However, a significant difference was detected in weight gain between the NiO group rats and the rats in the group where RES + NiO was administered together, compared to the control group rats (*p* < 0.05). However, no significant difference was observed between the NiO group and the RES + NiO group (*p* < 0.05). When evaluated in terms of kidney weights, no significant difference was observed between the control group and the NiO and RES + NiO groups of rats (*p* < 0.05).

**Table 3:** Effects of NiO and RES on the body and organ weights in rats

Parameters	Groups			
	Control	RES	NiO	RES + NiO
Body weight initial (g)	175.35 ± 5.75^a^	189 ± 12.81^a^	244 ± 15.17^a^	243 ± 18.67^a^
Body weight final (g)	241 ± 10.24^a^	252.5 ± 13.05^a^	265.2 ± 18.38^a^	277.8 ± 17.84^a^
Body weight change (%)	37.51 ± 5.77^a^	33.64 ± 4.02^a^	8.63 ± 1.39^,b^	14.43 ± 1.86^,b^
Absolute right kidney weight (g)	1.27 ± 0.08^a^	1.26 ± 0.11^a^	1.28 ± 0.11^a^	1.25 ± 0.1^a^
Relative right kidney weight (g)	0.5 ± 0.01^a^	0.5 ± 0.03^a^	0.48 ± 0.04^a^	0.45 ± 0.03^a^
Absolute left kidney weight (g)	1.26 ± 0.11^a^	1.24 ± 0.1^a^	1.3 ± 0.08^a^	1.27 ± 0.05^a^
Relative left kidney weight (g)	0.5 ± 0.01^a^	0.49 ± 0.02^a^	0.49 ± 0.04^a^	0.46 ± 0.02^a^

Results are given as mean ± SD (*n* = 6). Results are accepted as statistically significant when the *p* < 0.05. Different letters given as superscripts in each row indicate significance between groups

### Bioaccumulation of nickel

**Fig. 1:**
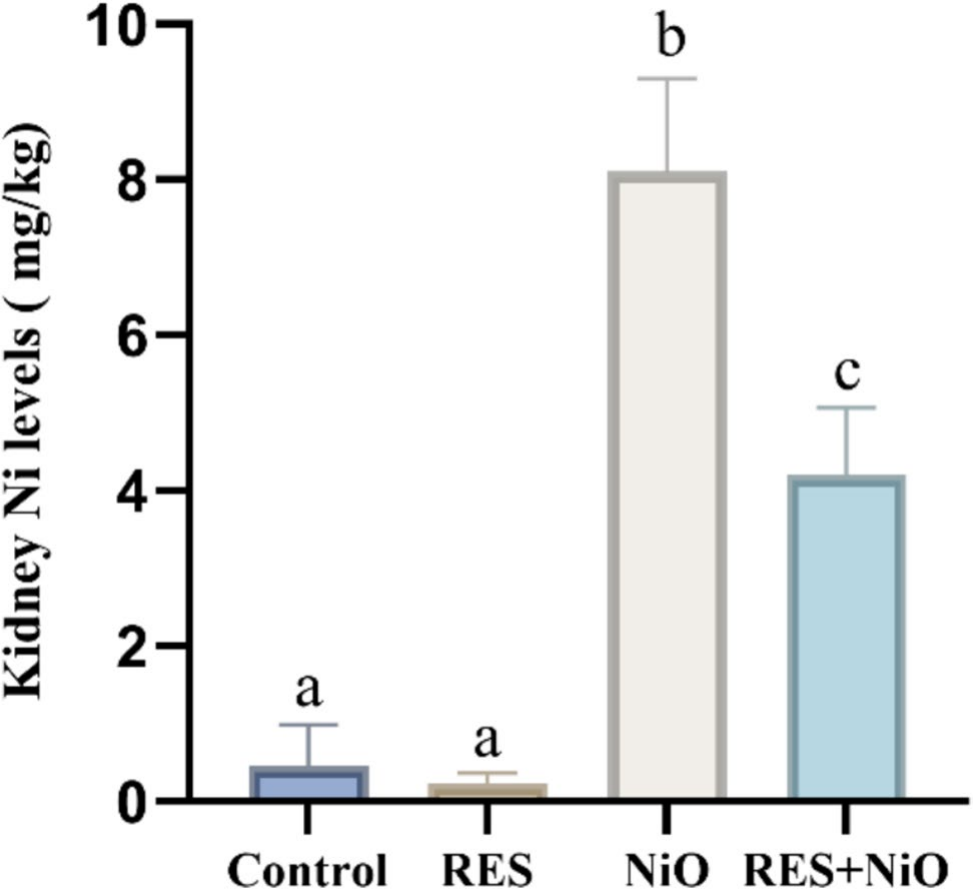
Measurement of nickel levels and statistical analysis between experimental groups. Results are given as mean ± SD (*n* = 6). Results are accepted as statistically significant when the *p* < 0.05. Different letters given as superscripts in each row indicate significance between groups

Figure 1 shows the level of nickel accumulated in the kidney tissues of all experimental groups. Compared to the control and RES groups, significant nickel accumulation was detected in the kidney tissue of rats in the NiO-treated group (*p* < 0.05). A significant decrease in nickel accumulation was observed in the kidney tissue of rats in the RES + NiO-treated group compared to the nickel accumulation in the NiO-treated group (*p* < 0.05).

### Renal function markers

As shown in Table 4, the urea, creatinine, and uric acid levels in the group of rats treated with NiO showed a significant increase compared to the control group (*p* < 0.05). It was found that resveratrol administered together with nickel oxide significantly reduced the urea, creatinine, and uric acid levels in the group of rats treated with nickel oxide alone (*p* < 0.05).

**Table 4:** Effects of NiO and RES on renal function parameters of rats

Parameters	Groups			
	Control	RES	NiO	RES + NiO
Creatinine (mg/dl)	0.67 ± 0.03^a^	0.66 ± 0.01^a^	0.77 ± 0.01^b^	0.73 ± 0.01^c^
Urea (mg/dl)	24.13 ± 0.82^a^	23.29 ± 0.73^a^	33.07 ± 0.82^b^	28.76 ± 0.67^c^
Uric acid (mg/dl)	1.53 ± 0.03^a^	1.52 ± 0.05^a^	1.91 ± 0.04^b^	1.71 ± 0.04^c^

Results are given as mean ± SD (*n* = 6). Results are accepted as statistically significant when the *p* < 0.05. Different letters given as superscripts in each row indicate significance between groups

### Oxidative stress parameters

**Fig. 2:**
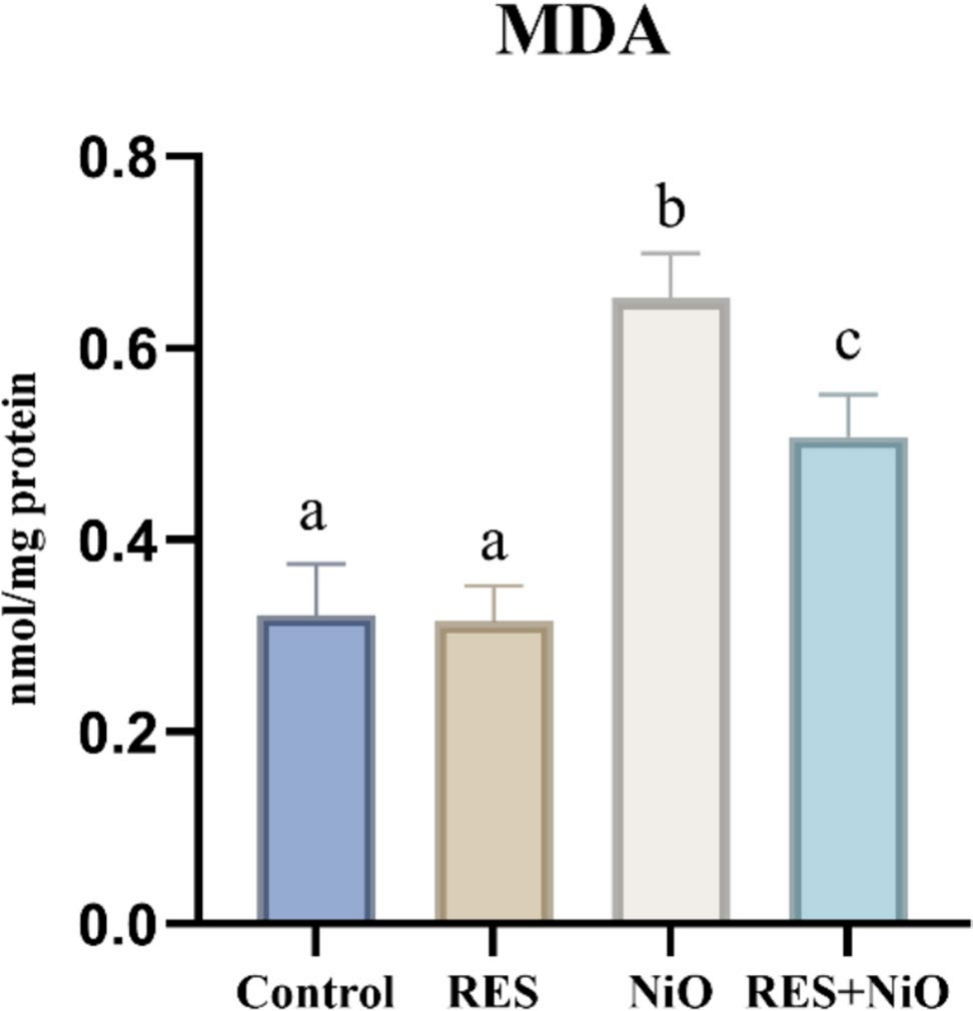
Measurement of MDA levels and statistical analysis between experimental groups. Results are given as mean ± SD (*n* = 6). Results are accepted as statistically significant when the *p* < 0.05. Different letters given as superscripts in each row indicate significance between groups

After administering NiO and RES to rats in the study, the MDA level, which is the end product of lipid peroxidation, was evaluated, and the obtained data are shown in Fig. 2. Compared to the control group, renal MDA levels were significantly elevated in the NiO-treated group (*p* < 0.05). In contrast, no significant difference was detected between the control and RES groups. RES treatment significantly reduced the NiO-induced increase in MDA, bringing it closer to the control group values (*p* < 0.05).

**Fig. 3:**
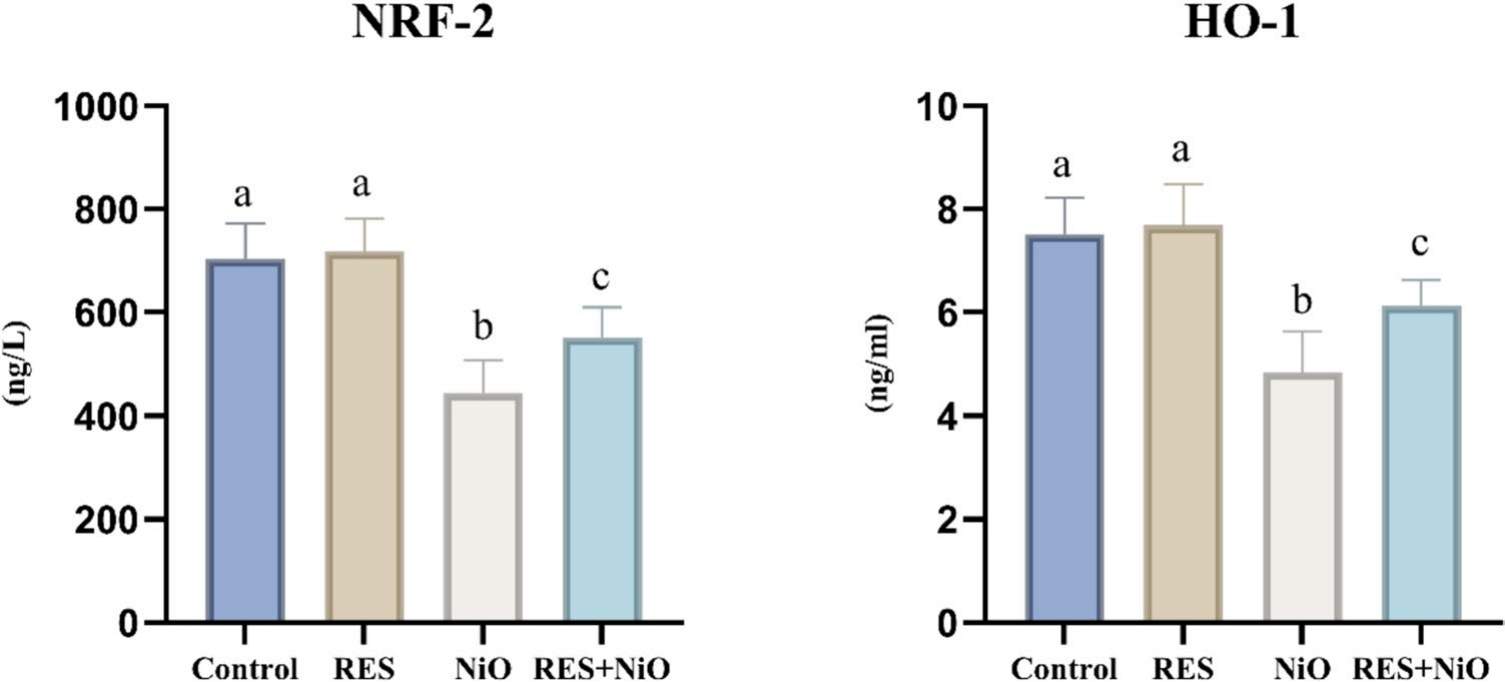
Measurement of Nrf2 and HO-1 levels and statistical analyses between experimental groups. Results are given as mean ± SD (*n* = 6). Results are accepted as statistically significant when the *p* < 0.05. Different letters given as superscripts in each row indicate significance between groups

The Nrf2 level regulating the expression of antioxidant activities and HO-1 enzyme activity is shown in Fig. 3. In the NiO-treated group, the Nrf2 level and HO-1 activity were significantly reduced compared to the control group (*p* < 0.05). In the group where RES and NiO were applied together, a significant increase in Nrf2 and HO-1 levels was detected (Fig. 3) (*p* < 0.05).

**Fig. 4:**
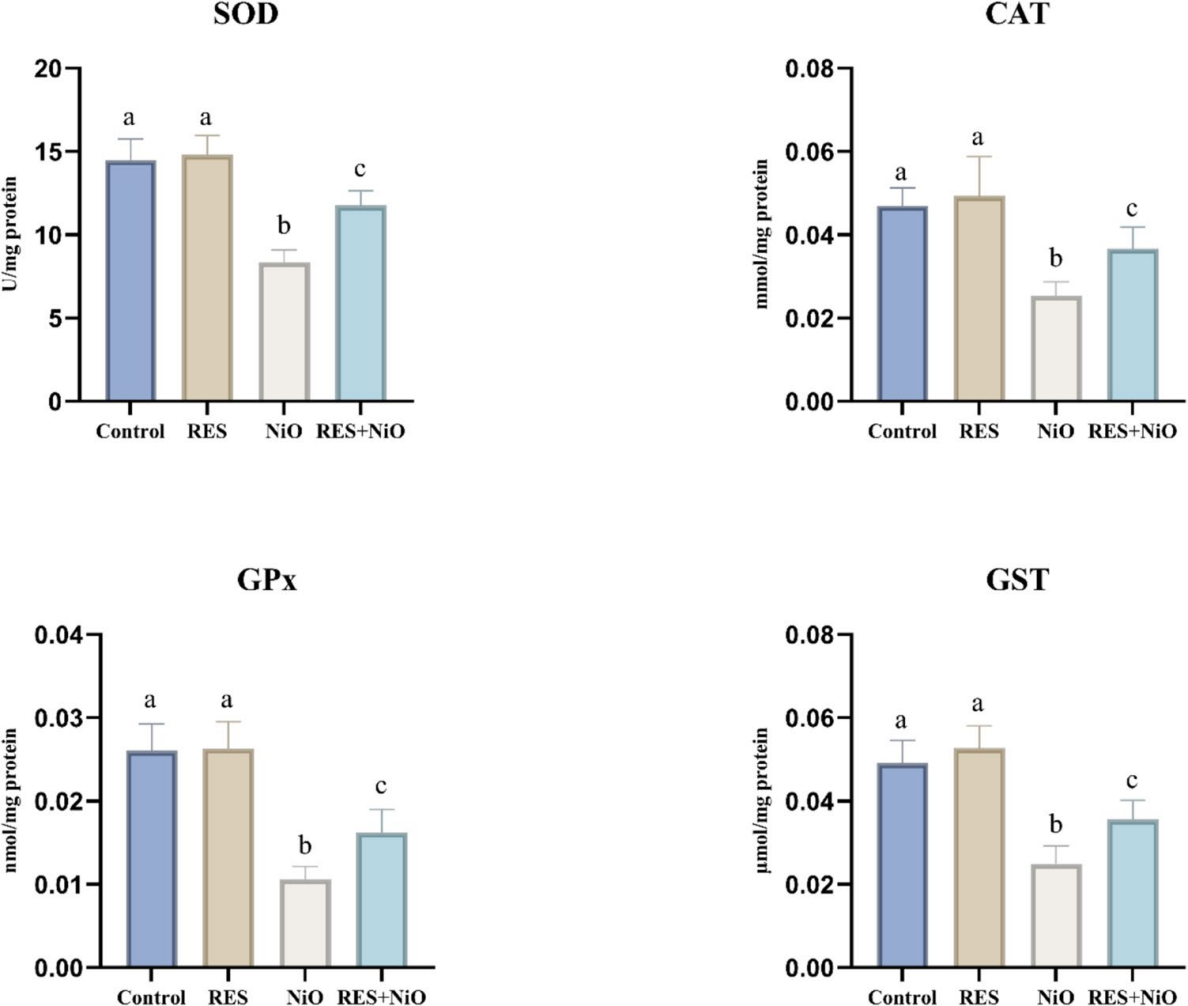
Measurement of SOD, CAT, GPx, and GST activities and statistical analysis between experimental groups. Results are given as mean ± SD (*n* = 6). Results are accepted as statistically significant when the *p* < 0.05. Different letters given as superscripts in each row indicate significance between groups

When renal tissue was evaluated for SOD, CAT, GPx, and GST antioxidant activities, a significant decrease was observed in the NiO-treated group compared to the control group (*p* < 0.05) (Fig. 4). RES supplementation, on the other hand, resulted in a significant increase in the parameters studied (*p* < 0.05) (Fig. 4).

**Fig. 5:**
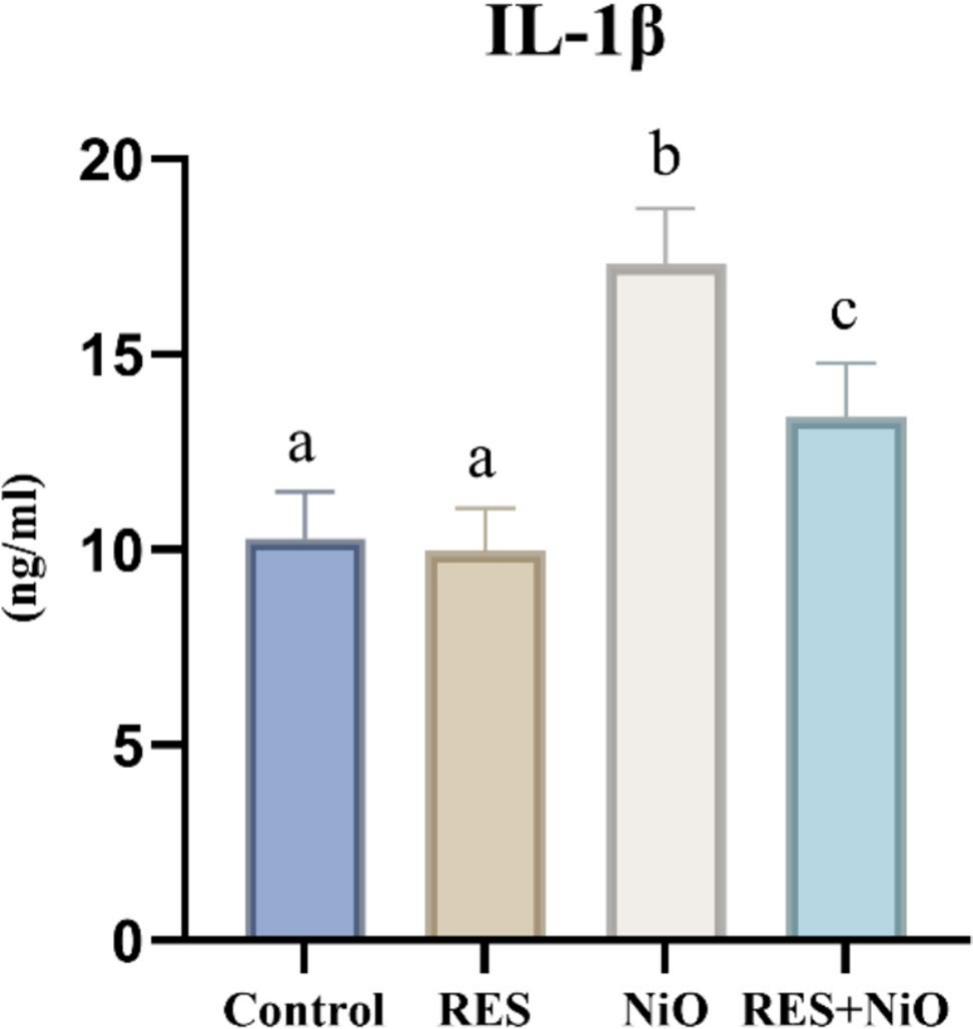
Measurement of IL-1β levels and statistical analysis between experimental groups. Results are given as mean ± SD (*n* = 6). Results are accepted as statistically significant when the *p* < 0.05. Different letters given as superscripts in each row indicate significance between groups

**Fig. 6:**
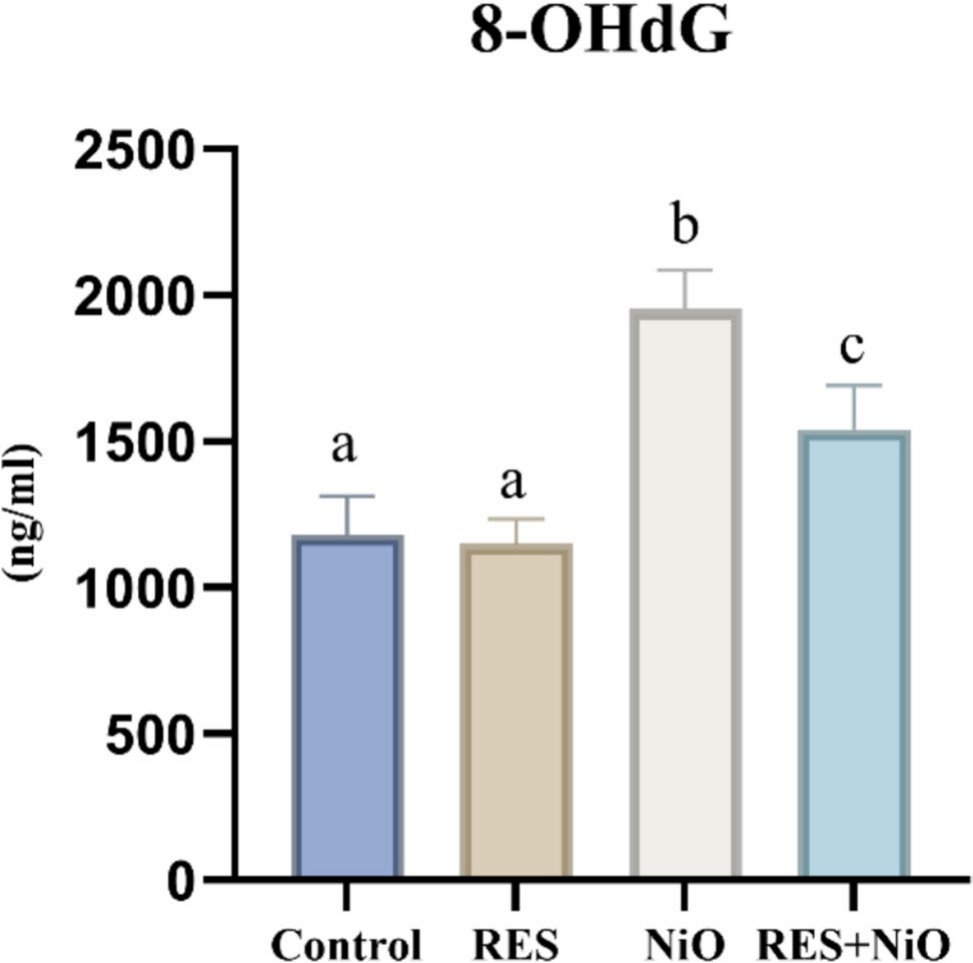
Measurement of 8-OHdG levels and statistical analysis between experimental groups. Results are given as mean ± SD (*n* = 6). Results are accepted as statistically significant when the *p* < 0.05. Different letters given as superscripts in each row indicate significance between groups

The IL-1β level in renal tissue was significantly elevated in the NiO-treated group compared to the control group (*p* < 0.05), while the inflammation level increased by RES supplementation decreased (*p* < 0.05) (Fig. 5). The level of 8-OHdG, which is frequently used to indicate oxidative DNA damage, was investigated in kidney tissues, and a significant increase was observed in the NiO-treated group compared to the control group (*p* < 0.05). In the group treated with NiO together with RES, the 8-OHdG level decreased dramatically (*p* < 0.05) (Fig. 6).

### Real-time PCR assessment

**Fig. 7:**
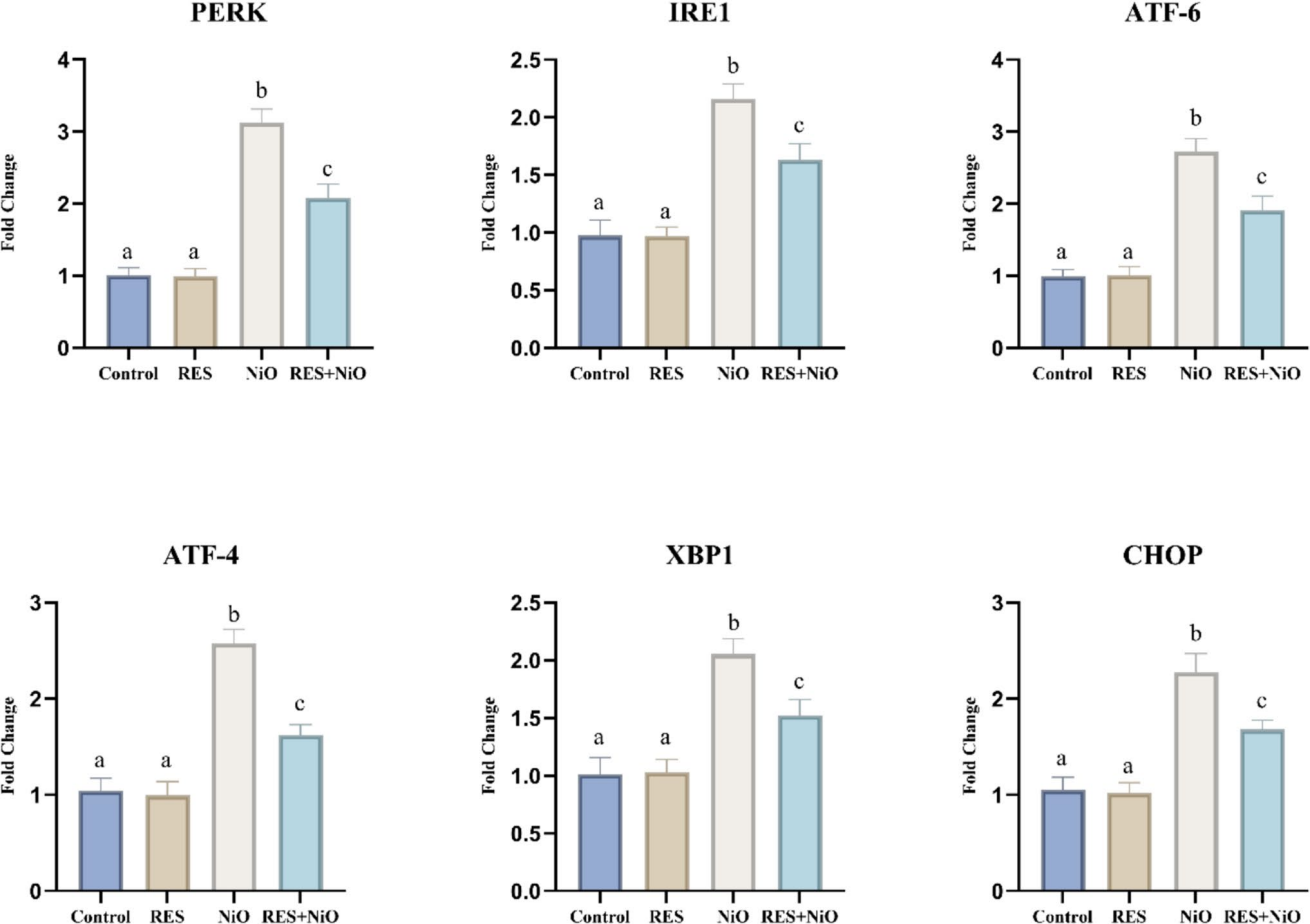
Relative mRNA transcript levels of PERK, ATF-6, ATF-4, IRE1, XBP1, and CHOP and statistical analysis among experimental groups. Results are given as mean ± SD (*n* = 6). Results are accepted as statistically significant when the *p* < 0.05. Different letters given as superscripts in each row indicate significance between groups

The relative mRNA transcription levels of PERK, ATF-6, ATF-4, IRE1, XBP1, and CHOP, genes, which are important markers of ER stress in renal tissue, are presented in Fig. 7. NiO application upregulated the mRNA transcription levels of PERK, ATF-6, ATF-4, IRE1, XBP1, and CHOP in renal tissue (*p* < 0.05). On the other hand, RES application dramatically reduced the endoplasmic reticulum stress markers induced by NiO application (*p* < 0.05) (Fig. 7).

### Immunohistochemical assessment

**Fig. 8:**
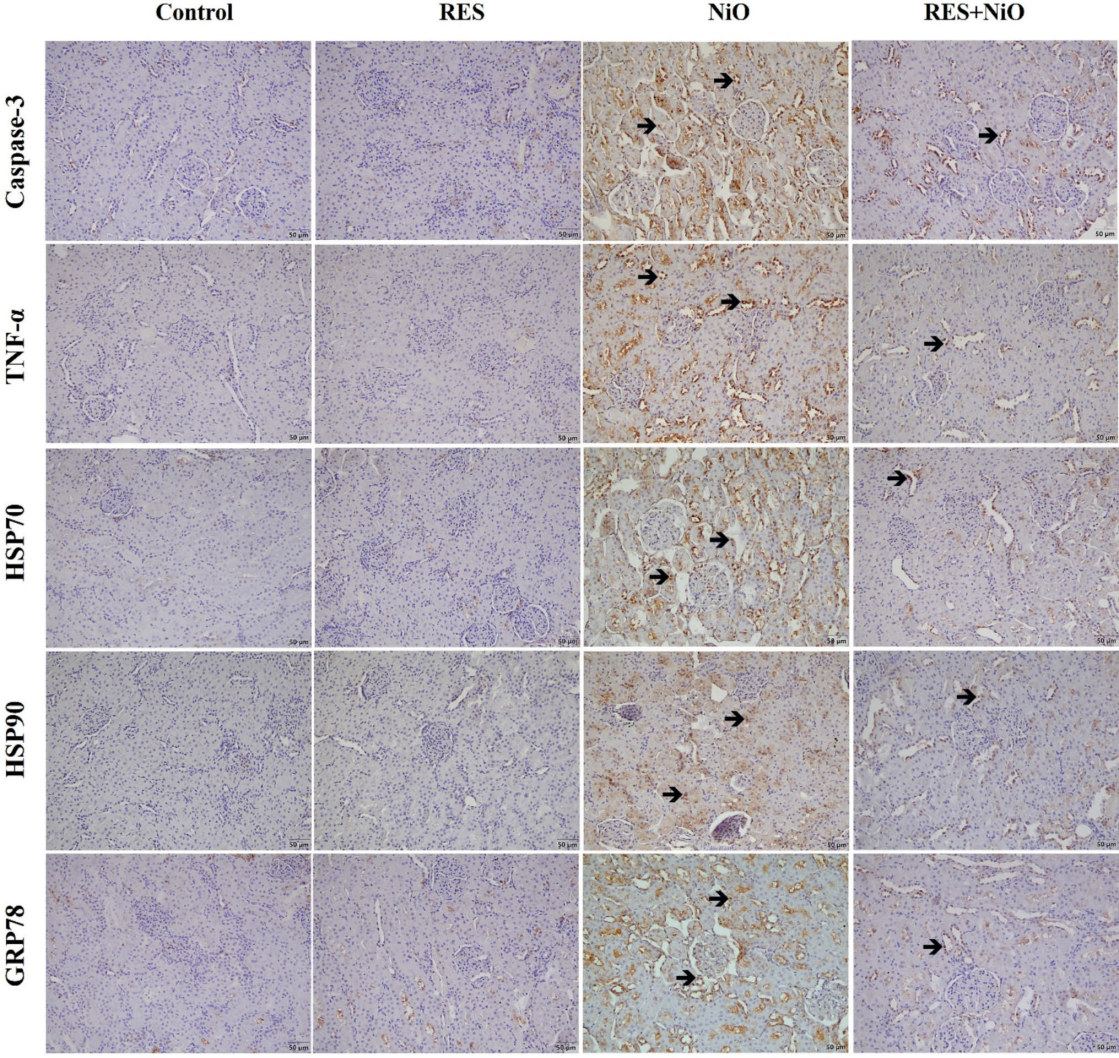
Immunohistochemical staining of caspase-3, TNF‐α, HSP70, HSP90, and GRP78 in kidney tissues. Control and RES groups showed weak staining for Caspase-3, TNF‐α, HSP70, HSP90, and GRP78, while NiO and RES + NiO groups showed increased expression compared to control and RES groups, immunopositive cell (). Scale bar: 50 μm

**Fig. 9:**
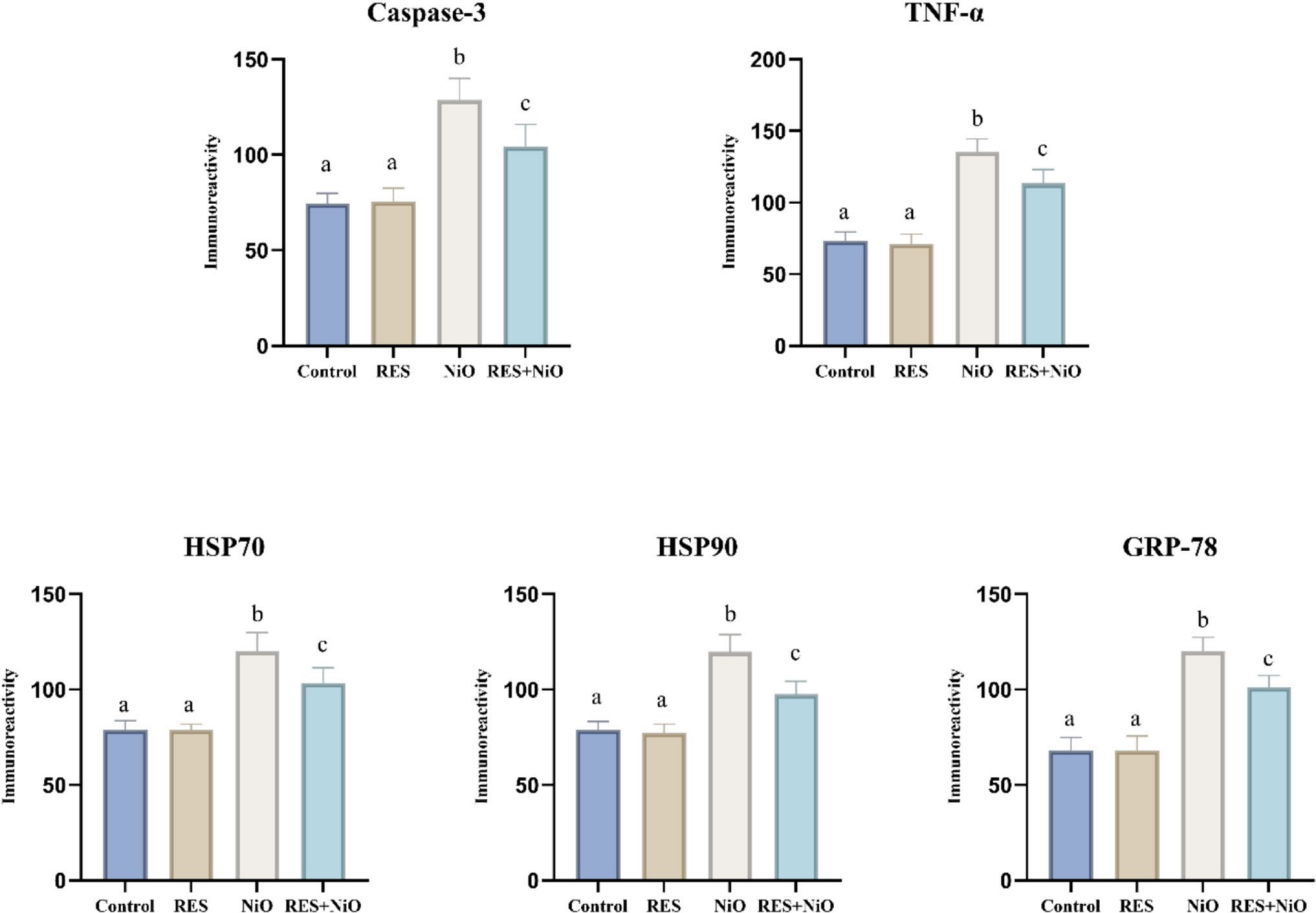
Analysis of immunoreactive density in the kidney for caspase-3, TNF‐α, HSP70, HSP90, GRP78. Results are accepted as statistically significant when the *p* < 0.05. Different letters given as superscripts in each row indicate significance between groups

To evaluate changes in the expression of caspase-3, TNF-α, HSP70, HSP90, and GRP78 expression levels were evaluated using immunohistochemical staining, and the number of positive cells for caspase-3, TNF-α, HSP70, HSP90, and GRP78 expression was significantly increased in the NiO-treated group compared to the control group (Fig. 8). In the group treated with NiO together with RES, the number of immunopositive cells decreased significantly compared to the NiO-treated group (Fig. 8). The intergroup immunoreactivity evaluation of the parameters examined in the kidney tissue is shown in Fig. 9 (*p* < 0.05).

### Histological assessment

**Fig. 10:**
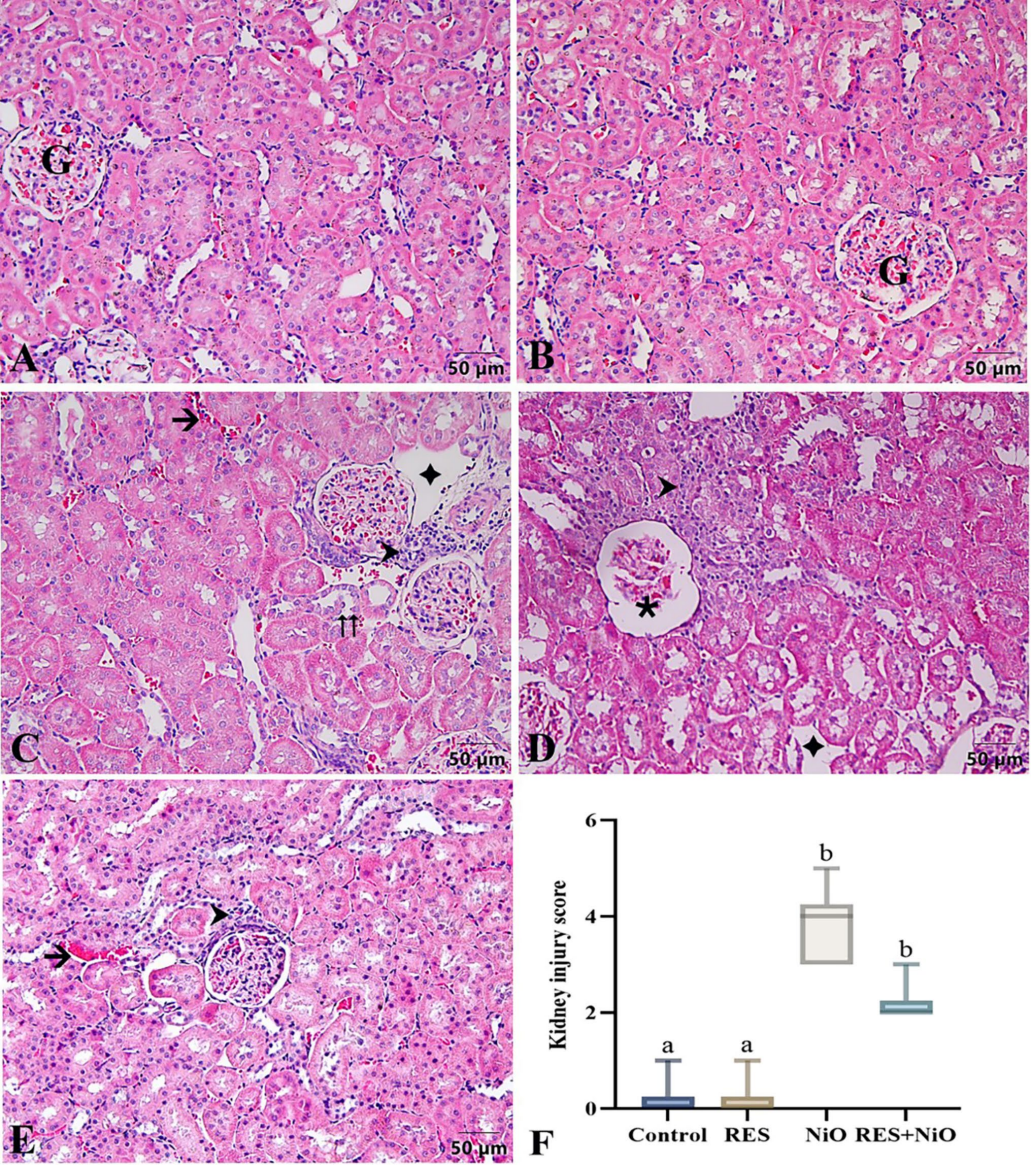
H&E image of kidney tissue. **A** Control and **B** RES groups. The glomerulus (**G**) and tubules appear to have a normal histological structure. **(C–D)** NiO group showed tubular degeneration (  ), inflammatory cell infiltration (  ), hemorrhage (  ), glomerular degeneration (  ) and edema (  ) in kidney. **E** RES + NiO group showed inflammatory cell infiltration (  ), hemorrhage (  ) in kidney. **F** Statistical analysis of kidney injury scores. Scale bar = 50 μm, *p* < 0.05

The glomerular and tubular structures in the kidney tissues of the control group and resveratrol group rats exhibit a normal histological appearance (Fig. 10A and B). In the NiO group, pathological lesions such as glomerular degeneration, tubular degeneration, hemorrhage, mononuclear cell infiltration, and edema were observed (Fig. 10C and D). In the RES + NiO group, the observed pathologies were milder than those in the NiO group, with hemorrhage and mononuclear cell infiltration observed (Fig. 10E). The histological scoring table of kidney tissue is given in Fig. 10F.

## Discussion

Toxicological studies show that renal toxicity causes direct tissue damage, inflammation, disruption of renal function, and changes in blood circulation (Al-Naimi et al. 2019). Among the basic functions of the kidney, plasma infiltration plays a crucial role in the secretion of erythropoietin and renin hormones, fluid homeostasis, and excretion of end products, but xenobiotics can impair the functioning of the kidney by disrupting the physiology and morphology of the kidney tissue (Rana 2021; Alhazmi et al. 2024). In the last few years, it has been known that exposure to heavy metals such as nickel leads to major health problems and that it is important to elucidate the mechanism underlying this toxicity (Hassanen et al. 2025). The LD_50_ dose of nickel oxide has been reported to be higher than 11,000 mg/kg in previous studies, and we would like to point out that the dose used in the current study is well below the LD_50_ dose (Henderson et al. 2012). Kidney tissue, which is very sensitive to particle toxicity along with its importance in the excretion of substances, is an organ where nickel metal accumulates (Abdulqadir and Aziz 2019a; Rana 2021). Resveratrol is a natural antioxidant and is an effective scavenger for radicals produced by lipid peroxidation, hydroxyls, and superoxide in cells (Hafez et al. 2025). In addition to scavenging free radicals, various studies suggest that resveratrol (RES) exhibits antioxidant effects not only directly through free radical scavenging but also through metal chelation. Due to its hydroxyl group, it coordinates redox-active ions such as Fe^2^⁺ and Cu^2^⁺, limiting their participation in Fenton-type reactions and reducing the formation of reactive oxygen species (Belguendouz et al. 1997; Dias and Nikolaou 2011; Zeng et al. 2024). As a result, it acts as a powerful metal chelator, enzyme regulator, and radical scavenger (de Oliveira et al. 2021).

Monitoring changes in body weights and organ weights is one of the important parameters in toxicological studies (Karaboduk et al. 2024b). In previous studies using nickel oxide, it was reported that there was a decrease in weight gain in rats (Singh et al. 2021, 2022a). In the current study, it was observed that NiO-treated rats had decreased weight gain, while RES supplementation caused weight gain. However, no change was observed in organ weights.

Dose-dependent metal accumulation in organs has been shown in many studies (Dumala et al. 2019; Ali et al. 2021). Similarly, repeated dose rat studies have shown that nickel accumulations translocate to organs such as the liver, kidney and testes via the gastrointestinal system, resulting in histopathological, biochemical, and cellular damage (Ali et al. 2021; Singh et al. 2021, 2022a). In the current study, it was observed that the nickel accumulation in the NiO-applied group increased significantly compared to the control and resveratrol groups, while it decreased significantly in the resveratrol-nickel-applied group. This may be attributed to the chelating property of resveratrol.

The concentration of creatine, urea, and uric acid serves as key biomarkers for assessing renal functional capacity. Changes in their levels during their excretion in the kidney are indicators of renal diseases and have always been important parameters used to measure kidney functions (Adiguzel and Kalender 2020). It has been reported in earlier research that nickel increases creatinine, urea, and uric acid levels in rat kidneys (Abdulqadir and Aziz 2019b; Ali et al. 2021; Singh et al. 2022b). In the current study, it was observed that NiO application increases these parameters. This can be attributed to the accumulation of nickel in the kidney tissue and the deterioration of the filtration structure (Dumala et al. 2018). Likewise, the histological changes observed in the current study support this situation. It has been observed that RES application is effective in reducing renal damage and reduces urea, creatinine, and uric acid levels. Similarly, Hu et al. (2017) reported that heavy metal application in rats increased urea and creatinine values in kidney tissue, while RES application alleviated kidney damage and approached normal values.

**Fig. 11:**
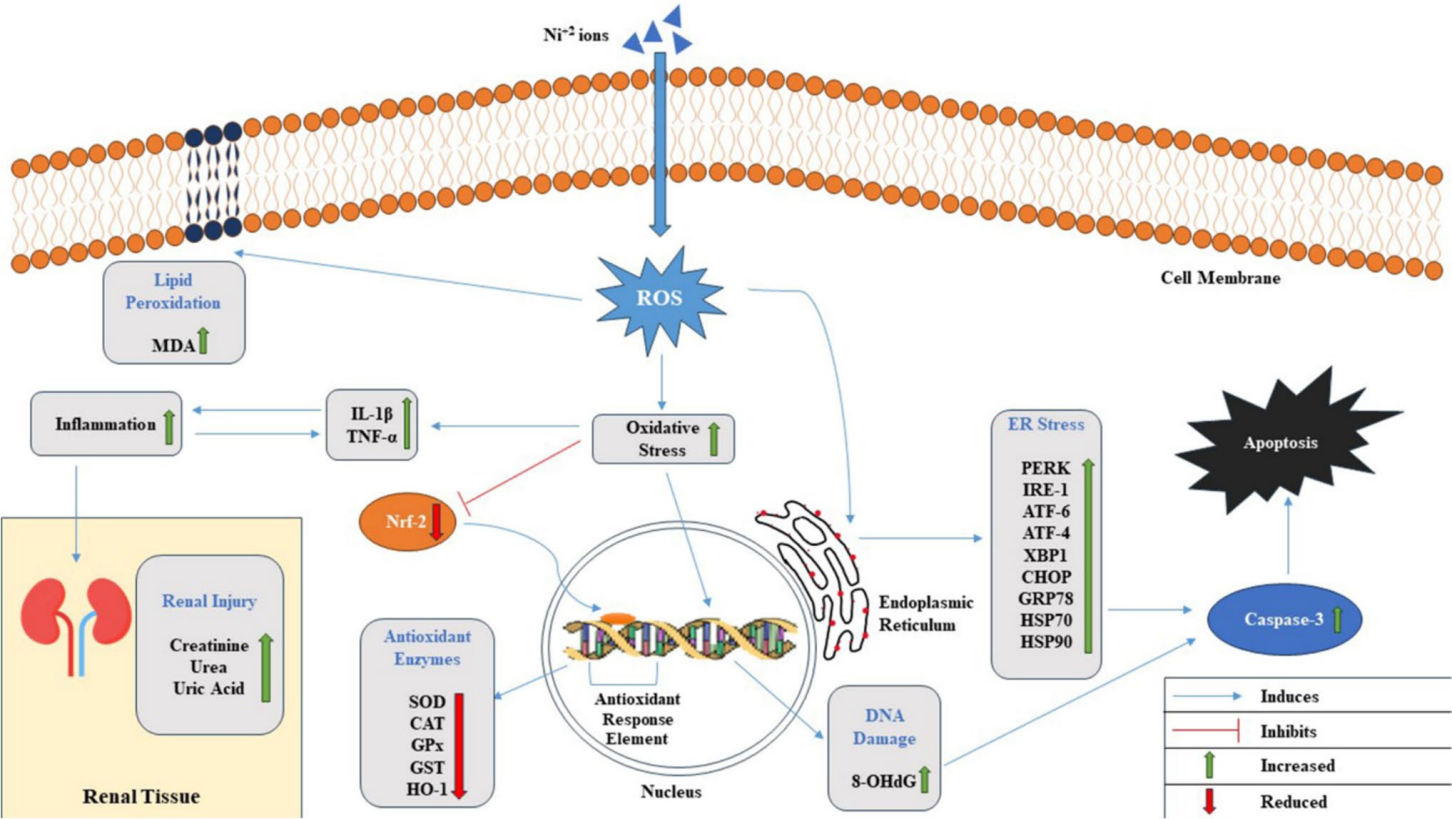
Schematic diagram illustrating the toxic mechanism of nickel

Excessive ROS increases lead to a decline in antioxidant capacity, causing oxidative stress, which in turn leads to progressive oxidative damage and tissue injury (Uzunhisarcikli et al. 2023). The ROS-induced toxic mechanism of nickel is shown in (Fig. 11). Oxidative stress is a direct contributor to the pathogenesis of numerous diseases. It elevates lipid peroxidation by directly targeting lipids, a primary constituent of the cell membrane (Kalender et al. 2019; Kankılıç et al. 2024). MDA, the final product of lipid peroxidation, is formed by the peroxidation of unsaturated fatty acids in the cell membrane and is shown to be an indirect indicator of tissue and organ damage (Adiguzel et al. 2024). Previous studies suggest that metallic nickel increases MDA levels by increasing ROS in tissues depending on the dose and duration (Singh et al. 2022b; Karaboduk et al. 2024a). In this study, MDA levels were found to have significantly increased in the renal tissue of the NiO group. This suggests that this is a result of lipid peroxidation caused by an increase in reactive oxygen species (ROS) in the kidney tissue of the NiO group. RES application significantly reduced the increase in LPO. RES, as a natural metal chelator, may protect cell membranes from lipid peroxidation with its hydroxyl, superoxide, and nitric oxide scavenging properties (AlBasher et al. 2020). Previous studies also suggest that RES reduces MDA levels (Hafez et al. 2025; Elewa et al. 2025).

Nrf2 is a primary transcription factor activated by excessive oxidative stress and used in many redox reactions (Akarsu et al. 2024). In addition to regulating the physiological response to oxidative stress, research has shown that Nrf2 has anti-inflammatory property by increasing the transcription of antioxidant genes (Sethi et al. 2025). Nrf2 is abundant in the cytoplasm but is transported to the nucleus during oxidative stress caused by increased ROS (Shaw and Chattopadhyay 2020). Upon nuclear translocation, Nrf2 binds to the Antioxidant Response Element (ARE). This binding activates a cellular defense pathway dependent on Nrf2, which upregulates enzymes like heme oxygenase-1 (HO-1) and glutamyl (Sethi et al. 2025). Previous research has demonstrated Heme oxygenase (HO-1) to have anti-inflammatory, antioxidant, and antiapoptotic properties and is critically important in oxidative stress, inflammation, and disease progression (O’Rourke et al. 2024). Studies have also reported that HO-1 improves mitochondrial function by reducing ROS (Even et al. 2018). Antioxidants such as SOD, CAT, GPx, and GST are known as primary antioxidants in cellular defense, and potential changes in their activities during oxidative stress promote oxidative damage (Karaboduk and Kalender 2021; Fidan et al. 2024). SOD is the first step in the cell against free radicals, catalyzing the superoxide radical to molecular oxygen and H_2_O_2_. CAT, on the other hand, decomposes the superoxide radicals converted to H_2_O_2_ by SOD into water and oxygen (Uzunhisarcikli et al. 2021; Dogan et al. 2025). GPx plays a role in the reduction of H_2_O_2_ to water, while GST is effective in the detoxification of xenobiotics and the reduction of H_2_O_2_ (Karaboduk et al. 2015; Apaydin et al. 2025). Previous studies have indicated that Ni induces oxidative stress and increases ROS in vivo studies, resulting in significant changes in antioxidant capacities (Alhazmi et al. 2024; El Brouzi et al. 2025). In the current study, NiO application caused a sharp reduction in Nrf2, HO-1, SOD, CAT, GPx, and GST enzyme activities, while resveratrol supplementation reduced NiO-induced toxicity and improved the parameters studied. The electron-donating properties of RES, thanks to its radical-scavenging power, may have reduced ROS levels by enhancing Nrf2/HO-1 activity (Elewa et al. 2025; Liu et al. 2025). Furthermore, the dissociation of Nrf2/Keap1 binding, coupled with increased Nrf2 expression and its localization to the nucleus, may have enhanced antioxidant defenses by increasing enzyme activities (Elewa et al. 2025).

Inflammation is a protective reaction to a variety of xenobiotic stimuli (Adiguzel et al. 2024). A linear correlation exists between oxidative stress and inflammation, primarily driven by enhanced levels of reactive oxygen species (ROS) (Ayhan et al. 2025). Excessive ROS production can trigger the activation of NF-κB, a key transcription factor that regulates the expression of numerous pro-inflammatory genes (Rana et al. 2020). As a marker of inflammatory damage, NF-κB plays a central role in cytokine activation and immune responses. Upon activation, NF-κB translocates to the nucleus and induces the expression of pro-inflammatory cytokines, such as TNF-α and IL-1β, which are critical in initiating and sustaining inflammation (Şimşek et al. 2023). While IL-1β is one of the proinflammatory cytokines and mediates the release of numerous cytokines, TNF-α functions to activate immune cells by stimulating them during inflammation (Xu et al. 2021; El Brouzi et al. 2025; Ayhan et al. 2025). Previous studies suggest that Ni applications increase inflammation (Karaboduk et al. 2024a; Hassanen et al. 2025). In the current study, NiO elevated inflammation in rat kidney tissue. RES application reduced the inflammation caused by NiO. It has been reported that RES regulates nuclear factor κB (NFκB) through properties such as ROS scavenging and hydrogen peroxide suppression, thereby lowering the expression of proinflammatory cytokines IL-1β and TNF-α (Abdelrahman et al. 2022).

8-OHdG is an often-used marker for detecting ROS-induced DNA damage during oxidative stress (Graille et al. 2020). The DNA base guanine is extremely vulnerable to ROS, and during oxidative stress, 8-OHdG oxidizes guanine, causing DNA damage (Kandemir et al. 2025). There have been studies in the past showing that NiO raises 8-OHdG levels (Adiguzel and Karaboduk 2024; Alhazmi et al. 2024). In the current study, oxidative stress caused by NiO increased the level of 8-OHdG in kidney tissues. RES support significantly reduced this level.

Apoptosis is an important mechanism for eliminating damaged, aged, and dysfunctional cells (Venkatesan et al. 2025). Caspase-3 is also a key factor in oxidative stress-mediated apoptosis due to increased ROS (Hassanen et al. 2025). Prior studies have suggested increased apoptosis in nickel-induced in vivo experimental models (Yildiz Deniz et al. 2024; Mehanna et al. 2025). In the current study, NiO treatment elevated caspase-3 expression in rat kidney tissue due to stress caused by excessive ROS production. It has been stated that RES increases antioxidant defense and prevents apoptosis against environmental toxins and increased ROS (Yuluğ et al. 2013). In this research, the RES application significantly reduced caspase-3 expression, indicating that RES plays an active role in caspase-3 modulation.

The endoplasmic reticulum (ER) is an essential organelle that plays critical roles in protein synthesis, folding, and maturation, as well as in the maintenance of cellular homeostasis and xenobiotic detoxification (Varışlı et al. 2023). During infections, calcium imbalance, or other physiological and pathological conditions, the accumulation of unfolded or misfolded proteins within the ER lumen increases, leading to ER stress. To restore ER function, cells activate the unfolded protein response (UPR), a highly conserved adaptive signaling pathway (Cakmak et al. 2023). Heat shock proteins (HSPs) constitute a family of molecular chaperones classified according to their functions, including HSP60, HSP70, HSP90, and HSP110. Under normal physiological conditions, their expression levels are low; however, exposure to cellular stress markedly induces their expression (Kandil et al. 2024). Among these, HSP70 plays a central role in ensuring proper protein folding, whereas HSP90 is involved in maintaining proteins in their functional conformations (Akin et al. 2025). In addition, glucose-regulated protein 78 (GRP78) is a key ER-resident chaperone that regulates protein folding and serves as a critical signaling regulator during ER stress (Yang et al. 2025b). Under endoplasmic reticulum (ER) stress, the unfolded protein response (UPR) is activated through three distinct transmembrane sensor proteins: protein kinase RNA-like ER kinase (PERK), inositol-requiring enzyme 1 (IRE1), and activating transcription factor 6 (ATF6) (Cakmak et al. 2023). Under normal physiological conditions, these sensors are bound to the ER chaperone glucose-regulated protein 78 (GRP78); however, ER stress leads to the dissociation of GRP78 and subsequent activation of these signaling pathways. Activation of PERK promotes the induction of activating transcription factor 4 (ATF4), whereas IRE1 activation results in the splicing and activation of X-box binding protein 1 (XBP1), thereby coordinating adaptive responses to restore ER homeostasis (Tatar and Tatar 2019; Varışlı et al. 2023). C/EBP homologous protein (CHOP) is expressed at very low levels under physiological conditions and contributes to the maintenance of cellular homeostasis; however, under prolonged or pathological ER stress, CHOP expression is markedly upregulated and functions as a key mediator of ER stress–induced apoptosis, facilitating the removal of irreversibly damaged cells (Hu et al. 2019). Accumulating evidence indicates that nickel exposure induces ER stress in both rats and mice, leading to the activation of UPR-associated signaling pathways and apoptotic responses (Chang et al. 2017; Zhou et al. 2023). In this study, NiO exposure significantly increased the expression of the chaperone proteins HSP70, HSP90, and GRP78, as well as the genes PERK, ATF6, IRE1, ATF4, XBP1, and CHOP in rat kidney tissue, indicating the induction of ER stress. Resveratrol (RES) supplementation markedly modulated the expression of these genes, demonstrating a protective effect against NiO-induced ER stress. This effect is thought to be related not only to the antioxidant properties of RES but also to its ability to suppress excessive UPR activation.

Exposure to heavy metals and other xenobiotics leads to severe histopathological changes in living tissues (Kalender et al. 2004; Apaydin et al. 2015; Karaboduk et al. 2025). Many previous studies have shown that in vivo applications of nickel metal, depending on dose and time, cause oxidative stress by promoting lipid peroxidation and inhibiting antioxidant capacity, resulting in serious changes in the histoarchitectural structures of organs (Pan et al. 2024; Alhazmi et al. 2024; Hassanen et al. 2025). In the study conducted by Sharma et al. (2024), it was reported that repeated nickel dosing caused a decrease in body weight gain and serious organ damage with oxidative stress in the kidney tissue. In addition, metal toxicity causes tubular degeneration in the kidney tissue, causing serious deterioration in the tubule transport system and reabsorption functions, thus altering kidney functions (Şimşek et al. 2023). In the current study, NiO administration was observed to produce serious histopathological effects in rat kidney tissue. These changes were paralleled by increases in urea, creatinine, and uric acid levels, key indicators of renal function and structural integrity, and by increased inflammation levels. RES treatment has been observed to improve NiO-induced renal histology. This is particularly reflected in the restoration of tubular and glomerular structures, improved biochemical values, and decreased inflammation.

## Conclusion

In this study, subchronic NiO exposure lowered oxidative stress in experimental animals by elevating lipid peroxidation and decreasing antioxidant activity. It also led to increased inflammation, apoptosis, and endoplasmic reticulum stress markers. Administering RES (a flavonoid with antioxidant and anti-inflammatory properties) to the animals resulted in improvements in altered renal biochemistry, inflammation, apoptosis, and a significantly altered histological appearance. These results suggest that natural compounds such as RES could be used as potential therapeutic agents against renal toxicity resulting from NiO administration.

## Data Availability

All data in the current study can be obtained from the corresponding author upon reasonable request.

## Abbreviations

IL-1β: Interleukin 1 beta
SOD: Superoxide dismutase
CAT: Catalase
GPx: Glutathione peroxidase
GST: Glutathione s-transferase
HO-1: Heme oxygenase-1
8-OHdG: 8-Hydroxy-2’-deoxyguanosine
TNF-α: Tumor necrosis factor–alpha
PERK: Protein kinase RNA-like ER kinase
ATF-6: Activating transcription factor 6
GRP78: 78 KDa glucose-regulated protein
ATF-4: Activating transcription factor 4
IRE1: Inositol-requiring enzyme 1
CHOP: C/EBP homologous protein
XBP1: X-box binding protein 1

## References

[CR1] Abdelrahman SA, Mahmoud AA, Abdelrahman AA, Samy W, Zaid Hassen Saleh E (2022) Histomorphological changes and molecular mechanisms underlying the ameliorative effect of resveratrol on the liver of silver nanoparticles-exposed rats. Ultrastruct Pathol 46(3):268–284 35471163 10.1080/01913123.2022.2067929

[CR2] Abdulqadir SZ, Aziz FM (2019) Nickel nanoparticles induced nephrotoxicity in rats: influence of particle size. Pak Vet J 39(4):548–552

[CR3] Abdulqadir SZ, Aziz FM (2019) Internalization and effects on cellular ultrastructure of nickel nanoparticles in rat kidneys. Int J Nanomed 14:3995–4005 10.2147/IJN.S200909 PMC6549414 31213811

[CR4] Adiguzel C, Kalender Y (2020) Bendiocarb-induced nephrotoxicity in rats and the protective role of vitamins C and E. Environ Sci Pollut Res 27:6449–6458 10.1007/s11356-019-07260-x 31873894

[CR5] Adiguzel C, Karaboduk H (2024) Biochemical, immunohistochemical, histopathological, and apoptotic evaluation of nickel oxide nanoparticle- and microparticle-induced testicular toxicity in male rats. ACS Omega 9(52):50910–50921 39758642 10.1021/acsomega.4c01005 PMC11696382

[CR6] Adiguzel C, Karaboduk H, Apaydin FG, Kalender S, Kalender Y (2023) Comparison of nickel oxide nano and microparticles toxicity in rat liver: molecular, biochemical, and histopathological study. Toxicol Res 12(5):741–750 10.1093/toxres/tfad062 PMC10615818 37915490

[CR7] Adiguzel C, Karaboduk H, Uzunhisarcikli M (2024) Protective role of melatonin against abamectin-induced biochemical, immunohistochemical, and ultrastructural alterations in the testicular tissues of rats. Microsc Microanal 30(5):962–977 39189879 10.1093/mam/ozae080

[CR8] Adiguzel C, Karaboduk H, Apaydın FG, Kalender Y (2025) Effects of quercetin on palladium chloride-ınduced endoplasmic reticulum stress, ınflammation, oxidative stress, and apoptosis in hepatorenal tissues. Microsc Microanal 31(4):ozaf077:1-18 10.1093/mam/ozaf077 40875569

[CR9] Aebi H (1984) Catalase in vitro. Methods Enzymol 105:121–126 6727660 10.1016/s0076-6879(84)05016-3

[CR10] Akarsu SA, İleritürk M, Küçükler S, Akaras N, Gür C, Kandemir FM (2024) Ameliorative effects of sinapic acid against vancomycin-induced testicular oxidative damage, apoptosis, inflammation, testicular histopathologic disorders and decreased epididymal sperm quality. Reprod Toxicol 129:108666 39059777 10.1016/j.reprotox.2024.108666

[CR11] Akin AT, Kaymak E, Ceylan T, Kuloglu N, Karabulut D, Toluk A (2025) Unveiling the protective potential of crocin in septic acute liver ınjury via assessment of TLR4/HGM1/NF-κB signaling pathway, oxidative stress and heat shock response. Cell Biochem Funct 43(2):e70058 39962902 10.1002/cbf.70058

[CR12] AlBasher G, Abdel-Daim MM, Almeer R, Ibrahim KA, Hamza RZ, Bungau S, Aleya L (2020) Synergistic antioxidant effects of resveratrol and curcumin against fipronil-triggered oxidative damage in male albino rats. Environ Sci Pollut Res 27(6):6505–6514 10.1007/s11356-019-07344-8 31873888

[CR13] Alhazmi AI, El-Refaei MF, Abdallah EA (2024) Protective effects of gallic acid against nickel-induced kidney injury: impact of antioxidants and transcription factor on the incidence of nephrotoxicity. Ren Fail 46(1):2344656 38685608 10.1080/0886022X.2024.2344656 PMC11062283

[CR14] Ali AAM, Mansour AB, Attia SA (2021) The potential protective role of apigenin against oxidative damage induced by nickel oxide nanoparticles in liver and kidney of male Wistar rat, *Rattus norvegicus*. Environ Sci Pollut Res 28:27577–27592 10.1007/s11356-021-12632-3 33515148

[CR15] Al-Naimi MS, Rasheed HA, Hussien NR, Al-Kuraishy HM, Al-Gareeb AI (2019) Nephrotoxicity: role and significance of renal biomarkers in the early detection of acute renal injury. J Adv Pharm Technol Res 10(3):95–99 31334089 10.4103/japtr.JAPTR_336_18 PMC6621352

[CR16] Apaydin FG, Kalender S, Bas H, Demir F, Kalender Y (2015) Lead nitrate induced testicular toxicity in diabetic and non-diabetic rats: protective role of sodium selenite. Braz Arch Biol Technol 58:68–74

[CR17] Apaydin FG, Kalender S, Bas H, Kalender Y (2025) Evaluation of hepatotoxicity and nephrotoxicity of fenitrothion on ultrastructural, ımmunohistochemical, histopathological, and biochemical changes: protective role of gallic acid. Microsc Microanal 31(4):ozaf068 40828939 10.1093/mam/ozaf068

[CR18] Ayhan H, Adiguzel C, Bayram KK, Akin AT, Apaydin FG, Kalender Y (2025) Effect of propolis supplementation on cadmium toxicity associated with renal and hepatic dysfunction in rats. J Trace Elem Med Biol 87:127587 39764897 10.1016/j.jtemb.2024.127587

[CR19] Belguendouz L, Frémont L, Linard A (1997) Resveratrol inhibits metal ion-dependent and independent peroxidation of porcine low-density lipoproteins. Biochem Pharmacol 53(9):1347–1355 9214696 10.1016/s0006-2952(96)00820-9

[CR20] Cakmak F, Kucukler S, Gur C, Comakli S, Ileriturk M, Kandemir FM (2023) Morin provides therapeutic effect by attenuating oxidative stress, inflammation, endoplasmic reticulum stress, autophagy, apoptosis, and oxidative DNA damage in testicular toxicity caused by ifosfamide in rats. Iran J Basic Med Sci 26(10):1227 37736509 10.22038/IJBMS.2023.71702.15580 PMC10510477

[CR21] Chang X, Liu F, Tian M, Zhao H, Han A, Sun Y (2017) Nickel oxide nanoparticles induce hepatocyte apoptosis via activating endoplasmic reticulum stress pathways in rats. Environ Toxicol 32(12):2492–2499 28945320 10.1002/tox.22492

[CR22] de Oliveira SA, Cerri PS, Sasso-Cerri E (2021) Impaired macrophages and failure of steroidogenesis and spermatogenesis in rat testes with cytokines deficiency induced by diacerein. Histochem Cell Biol 156:561–581 34515835 10.1007/s00418-021-02023-7 PMC8436873

[CR23] Dias K, Nikolaou S (2011) Does the combination of resveratrol with Al (III) and Zn (II) ımprove its antioxidant activity? Nat Prod Commun 6(11):1934578X1100601 22224286

[CR24] Diaz-Gerevini GT, Repossi G, Dain A, Tarres MC, Das UN, Eynard AR (2016) Beneficial action of resveratrol: how and why? Nutrition 32(2):174–178 26706021 10.1016/j.nut.2015.08.017

[CR25] Dogan T, Yıldırım BA, Kapakin KAT, Kiliçliogli M, Senocak EA (2025) Protective effects of crocin against gentamicin-induced damage in rat testicular tissue: modulating the levels of NF-κB/TLR-4 and Bax/Bcl-2/caspase-3 signaling pathways. Food Chem Toxicol 200:115407 40127811 10.1016/j.fct.2025.115407

[CR26] Dumala N, Mangalampalli B, Kalyan Kamal SS, Grover P (2018) Biochemical alterations induced by nickel oxide nanoparticles in female Wistar albino rats after acute oral exposure. Biomarkers 23(1):33–43 28748734 10.1080/1354750X.2017.1360943

[CR27] Dumala N, Mangalampalli B, Kalyan Kamal SS, Grover P (2019) Repeated oral dose toxicity study of nickel oxide nanoparticles in Wistar rats: a histological and biochemical perspective. J Appl Toxicol 39(7):1012–1029 30843265 10.1002/jat.3790

[CR28] Duta-Bratu CG, Nitulescu GM, Mihai DP, Olaru OT (2023) Resveratrol and other natural oligomeric stilbenoid compounds and their therapeutic applications. Plants 12(16):2935–2935 37631147 10.3390/plants12162935 PMC10459741

[CR29] El Brouzi MY, Adadi N, Lamtai M, Boulahfa H, Zghari O, Fath N, Rezqaoui A, El Hamzaoui A, Njimat S, El Hessni A, Mesfioui A (2025) Effects of nickel bioaccumulation on hematological, biochemical, ımmune responses, neuroinflammatory, oxidative stress parameters, and neurotoxicity in rats. Biol Trace Elem Res. 10.1007/s12011-025-04528-x 39891830 10.1007/s12011-025-04528-x

[CR30] Elewa HS, Salama DA, Hikal MS, El hamid MFA, Eid MH, Khalil FM, Abdalrani MS, Abdelaal K, El-Tokhy AI (2025) Protective effects of resveratrol and naringenin against nonylphenol-induced oxidative stress in rats. AMB Express 15(1):7 39779659 10.1186/s13568-024-01788-z PMC11711685

[CR31] Even B, Fayad-Kobeissi S, Gagliolo JM, Motterlini R, Boczkowski J, Foresti R, Dagouassat M (2018) Heme oxygenase-1 induction attenuates senescence in chronic obstructive pulmonary disease lung fibroblasts by protecting against mitochondria dysfunction. Aging Cell 17(6):e12837 30341816 10.1111/acel.12837 PMC6260925

[CR32] Fidan EB, Bali EB, Apaydin FG (2024) Comparative study of nickel oxide and nickel oxide nanoparticles on oxidative damage, apoptosis and histopathological alterations in rat lung tissues. J Trace Elem Med Biol 83:127379 38171038 10.1016/j.jtemb.2023.127379

[CR33] Graille M, Wild P, Sauvain JJ, Hemmendinger M, Guseva Canu I, Hopf NB (2020) Urinary 8-OHdG as a biomarker for oxidative stress: a systematic literature review and meta-analysis. Int J Mol Sci 21(11):3743 32466448 10.3390/ijms21113743 PMC7313038

[CR34] Habig WH, Pabst MJ, Jakoby WB (1974) Glutathione S-transferases: the first enzymatic step in mercapturic acid formation. J Biol Chem 249(22):7130–7139 4436300

[CR35] Hafez MH, El-Kazaz SES, El-Neweshy MS, Shukry M, Ghamry HI, Tohamy HG (2025) Resveratrol mitigates heat stress-induced testicular injury in rats: enhancing male fertility via antioxidant, antiapoptotic, pro-proliferative, and anti-inflammatory mechanisms. Naunyn-Schmiedeberg’s Arch Pharmacol 398: 8359–8373 10.1007/s00210-024-03759-4 39792167

[CR36] Hassanen EI, Hassan NH, Mehanna S, Hussien AM, Ibrahim MA, Mohammed FF, Farroh KY (2025) Oral supplementation of curcumin-encapsulated chitosan nanoconjugates as an innovative strategy for mitigating nickel-mediated hepatorenal toxicity in rats. Naunyn-Schmiedeberg’s Arch Pharmacol 398: 8653–8668 10.1007/s00210-025-03799-4 PMC12263483 39836252

[CR37] Henderson RG, Durando J, Oller AR, Merkel DJ, Marone PA, Bates HK (2012) Acute oral toxicity of nickel compounds. Regul Toxicol Pharmacol 62(3):425–432 22333739 10.1016/j.yrtph.2012.02.002

[CR38] Hu J, Zhang BO, Du L, Chen J, Lu Q (2017) Resveratrol ameliorates cadmium induced renal oxidative damage and inflammation. Int J Clin Exp Med 10(5):7563–7572

[CR39] Hu H, Tian M, Ding C, Yu S (2019) The C/EBP homologous protein (CHOP) transcription factor functions in endoplasmic reticulum stress-induced apoptosis and microbial infection. Front Immunol 9:3083 30662442 10.3389/fimmu.2018.03083 PMC6328441

[CR40] Ibrahim MA, Albahlol IA, Wani FA, Tammam AAE, Kelleni MT, Sayeed MU, Mohamed AA (2021) Resveratrol protects against cisplatin-induced ovarian and uterine toxicity in female rats by attenuating oxidative stress, inflammation and apoptosis. Chem-Biol Interact 338:109402 33587916 10.1016/j.cbi.2021.109402

[CR41] Jeong M-J, Jeon S, Yu H-S, Cho W-S, Lee S, Kang D, Kim Y, Kim Y-J, Kim S-Y (2022) Exposure to nickel oxide nanoparticles induces acute and chronic inflammatory responses in rat lungs and perturbs the lung microbiome. Int J Environ Res Public Health 19(1):522–522 35010784 10.3390/ijerph19010522 PMC8744909

[CR42] Joyner NA, Romeu JGF, Kent B, Dixon DA (2024) The electronic structure of diatomic nickel oxide. Phys Chem Chem Phys 26(29):19646–19657 38957895 10.1039/d4cp01796j

[CR43] Kalender Y, Kalender S, Uzunhisarcikli M, Ogutcu A, Açikgoz F, Durak D (2004) Effects of endosulfan on B cells of Langerhans islets in rat pancreas. Toxicology 200(2–3):205–211 15212816 10.1016/j.tox.2004.03.017

[CR44] Kalender S, Apaydin FG, Kalender Y (2019) Testicular toxicity of orally administrated bisphenol A in rats and protective role of taurine and curcumin. Pak J Pharm Sci 32(3):1043–1047 31278718

[CR45] Kandemir O, Kucukler S, Comakli S, Gur C, İleriturk M (2025) Docetaxel-induced liver and kidney toxicity in rats can be alleviated by suppressing oxidative stress, endoplasmic reticulum stress, inflammation, apoptosis and autophagy signaling pathways after Silymarin treatment. Food Chem Toxicol 196:115202 39675460 10.1016/j.fct.2024.115202

[CR46] Kandil B, Kurtdede N, Bayraktaroglu AG (2024) Immunohistochemical localization and expression of heat shock proteins (HSP27, HSP60, HSP70, and HSP90) during the oestrous cycle, pregnancy, and lactation in rat ovaries. Acta Histochem 126(3):152157 38581753 10.1016/j.acthis.2024.152157

[CR47] Kankılıç NA, Küçükler S, Gür C, Akarsu SA, Akaras N, Şimşek H, İleritürk M, Kandemir FM (2024) Naringin protects against paclitaxel-induced toxicity in rat testicular tissues by regulating genes in pro-inflammatory cytokines, oxidative stress, apoptosis, and JNK/MAPK signaling pathways. J Biochem Mol Toxicol 38(7):e23751 38879801 10.1002/jbt.23751

[CR48] Karaboduk H, Kalender Y (2021) The effects of lead nitrate and mercury chloride on rat liver tissue. Fresenius Environ Bull 30(3):2368–2379

[CR49] Karaboduk H, Uzunhisarcikli M, Kalender Y (2015) Protective effects of sodium selenite and vitamin E on mercuric chloride-induced cardiotoxicity in male rats. Braz Arch Biol Technol 58(2):229–238

[CR50] Karaboduk H, Adiguzel C, Apaydin FG, Kalender S, Kalender Y (2024) Investigating the impact of different routes of nano and micro nickel oxide administration on rat kidney architecture, apoptosis markers, oxidative stress, and histopathology. J Mol Histol 55(5):675–686 38990468 10.1007/s10735-024-10221-5

[CR51] Karaboduk H, Adiguzel C, Apaydin FG, Uzunhisarcikli M, Kalender S, Kalender Y (2024) The ameliorative effect of Naringenin on fenamiphos induced hepatotoxicity and nephrotoxicity in a rat model: oxidative stress, inflammatory markers, biochemical, histopathological, immunohistochemical and electron microscopy study. Food Chem Toxicol 192:114911 39134134 10.1016/j.fct.2024.114911

[CR52] Karaboduk H, Adiguzel C, Uzunhisarcikli M, Apaydin FG, Kalender Y (2025) Melatonin mitigates abamectin-induced subacute hematotoxicity and hepato-renal toxicity in rats by regulating oxidative stress, inflammatory responses, and apoptosis. J Biochem Mol Toxicol 39(10):e70512 40976836 10.1002/jbt.70512

[CR53] Liu J, Han X, Zhang T, Tian K, Li Z, Luo F (2023) Reactive oxygen species (ROS) scavenging biomaterials for anti-inflammatory diseases: from Mechanism to Therapy. J Hematol Oncol Pharm 16(1):116 10.1186/s13045-023-01512-7 PMC10687997 38037103

[CR54] Liu L, Shi M, Wu Y, Hao J, Guo J, Li S, Dai P, Gao J (2025) Protective effects of resveratrol on honeybee health: mitigating pesticide-induced oxidative stress and enhancing detoxification. Pestic Biochem Physiol 210:106403 40262860 10.1016/j.pestbp.2025.106403

[CR55] Livak KJ, Schmittgen TD (2001) Analysis of relative gene expression data using real-time quantitative PCR and the 2− ΔΔCT method. Methods 25(4):402–408 11846609 10.1006/meth.2001.1262

[CR56] Lowry OH, Rosebrough NJ, Farr AL, Randall RJ (1951) Protein measurement with the Folin phenol reagent. J Biol Chem 193(1):265–275 14907713

[CR57] Lyons-Darden T, Blum JL, Schooley MW, Ellis M, Durando J, Merrill D, Oller AR (2023) An assessment of the oral and inhalation acute toxicity of nickel oxide nanoparticles in rats. Nanomaterials 13(2):261–261 36678015 10.3390/nano13020261 PMC9860552

[CR58] Manna I, Bandyopadhyay M (2019) Physicochemical perturbation of plants on exposure to metal oxide nanoparticle. In Tripathi DK et al (eds) Nanomaterials in plants, algae and microorganisms: concepts and controversies elsevier eBooks 2:323–352

[CR59] Marklund S, Marklund G (1974) Involvement of the superoxide anion radical in the autoxidation of pyrogallol and a convenient assay for superoxide dismutase. Eur J Biochem 47(3):469–474 4215654 10.1111/j.1432-1033.1974.tb03714.x

[CR60] Mehanna S, Hassan NH, Ibrahim MA, Mohammed FF, Hassanen EI (2025) Neurobehavioral and neuropathological alterations ınduced by nickel sulphate toxicity in rats: molecular mechanisms and prophylaxis with curcumin supplementation. Biol Trace Elem Res. 10.1007/s12011-025-04654-6 40531286 10.1007/s12011-025-04654-6 PMC12672646

[CR61] Noshy PA, Khalaf AAA, Ibrahim MA, Mekkawy AM, Abdelrahman RE, Farghali A, Tammam AA-E, Zaki AR (2022) Alterations in reproductive parameters and steroid biosynthesis induced by nickel oxide nanoparticles in male rats: the ameliorative effect of hesperidin. Toxicology 473:153208 35569531 10.1016/j.tox.2022.153208

[CR62] O’Rourke SA, Shanley LC, Dunne A (2024) The Nrf2-HO-1 system and inflammaging. Front Immunol 15:1457010 39380993 10.3389/fimmu.2024.1457010 PMC11458407

[CR63] Ohkawa H, Ohishi N, Yagi K (1979) Assay for lipid peroxides in animal tissues by thiobarbituric acid reaction. Anal Biochem 95(2):351–358 36810 10.1016/0003-2697(79)90738-3

[CR64] Paglia DE, Valentine WN (1987) Studies on the quantative and qualitative characterization of glutathione peroxidase. Transl Res 70:158–165 6066618

[CR65] Pan YL, Wu RZ, Fu Y, Xin R, Wu YH (2024) Protective effect of resveratrol on nickel-refining fumes-induced inflammatory damage. Cell Biochem Biophys 82(2):1121–1134 38589767 10.1007/s12013-024-01263-3

[CR66] Peng Y, Zheng X, Zhang S, Luo Z, Song L, Chen H, Yao X (2025) Advances in the activity of resveratrol and its derivatives in cardiovascular diseases. Arch Pharm 358(2):e2400865 10.1002/ardp.202400865 39956927

[CR67] Rana SVS (2021) Recent advances on renal toxicity of engineered nanoparticles-a review. J Toxicol Risk Assess 7:36

[CR68] Rana MN, Tangpong J, Rahman MA (2020) Xanthones protects leadinduced chronic kidney disease (CKD) via activating Nrf-2 and modulating NF-kB, MAPK pathway. Biochem Biophys Rep 21:100718 31886417 10.1016/j.bbrep.2019.100718 PMC6920509

[CR69] Sethi P, Mehan S, Khan Z, Maurya PK, Kumar N, Kumar A, Tiwari A, Sharma T, Das Gupta G, Narula AS, Kalfin R (2025) The SIRT-1/Nrf2/HO-1 axis: guardians of neuronal health in neurological disorders. Behav Brain Res 476:115280 39368713 10.1016/j.bbr.2024.115280

[CR70] Sharma M, Devi P, Kaushal S, Ul-Ahsan A, Mehra S, Budhwar M, Chopra M (2024) Cyto and genoprotective potential of tannic acid against cadmium and nickel co-exposure induced hepato-renal toxicity in BALB/c mice. Biol Trace Elem Res 202(12):5624–5636 38393487 10.1007/s12011-024-04117-4

[CR71] Shaw P, Chattopadhyay A (2020) Nrf2–ARE signaling in cellular protection: mechanism of action and the regulatory mechanisms. J Cell Physiol 235(4):3119–3130 31549397 10.1002/jcp.29219 PMC13482834

[CR72] Şimşek H, Küçükler S, Gür C, Akaras N, Kandemir FM (2023) Protective effects of sinapic acid against lead acetate-induced nephrotoxicity: a multi-biomarker approach. Environ Sci Pollut Res 30(45):101208–101222 10.1007/s11356-023-29410-y 37648919

[CR73] Singh M, Verma Y, Rana SVS (2021) Hepatotoxicity induced by nickel nano and microparticles in male rat: a comparative study. Toxicol Environ Health Sci 13:251–260

[CR74] Singh M, Verma Y, Rana SVS (2022) Attributes of oxidative stress in the reproductive toxicity of nickel oxide nanoparticles in male rats. Environ Sci Pollut Res 29(4):5703–5717 10.1007/s11356-021-15657-w 34424461

[CR75] Singh M, Verma Y, Rana SVS (2022) Nephrotoxicity of nickel nano and microparticles in rat-a comparative, time dependent study with special reference to antioxidant defence system. Inorg Nano-Met Chem 52(9):1335–1344

[CR76] Szymkowiak I, Marcinkowska J, Kucinska M, Regulski M, Murias M (2025) Resveratrol bioavailability after oral administration: a meta-analysis of clinical trial data. Phytother Res 39(1):453–464 39557444 10.1002/ptr.8379

[CR77] Tatar M, Tatar T (2019) Endoplazmik retikulum stresi ve ilişkili hastalıklar. Osmangazi J Med 41(3):294–303

[CR78] Uzunhisarcikli M, Apaydin FG, Bas H, Kalender Y (2021) Hepatoprotective effects of quercetin and curcumin against fipronil-induced hepatic injury in rats. Fresenius Environ Bull 30:9309–9321

[CR79] Uzunhisarcikli M, Apaydin FG, Bas H, Kalender Y (2023) The ameliorative effects of quercetin and curcumin against subacute nephrotoxicity of fipronil induced in Wistar rats. Toxicol Res 12(3):493–502 10.1093/toxres/tfad034 PMC10311137 37397921

[CR80] Valletta A, Iozia LM, Leonelli F (2021) Impact of environmental factors on stilbene biosynthesis. Plants 10(1):90 33406721 10.3390/plants10010090 PMC7823792

[CR81] Varışlı B, Caglayan C, Kandemir FM, Gür C, Ayna A, Genç A, Taysı S (2023) Chrysin mitigates diclofenac-induced hepatotoxicity by modulating oxidative stress, apoptosis, autophagy and endoplasmic reticulum stress in rats. Mol Biol Rep 50(1):433–442 36344803 10.1007/s11033-022-07928-7

[CR82] Venkatesan K, Sivadasan D, Abderrahmen Al Weslati M, Gayasuddin Mouid M, Goyal M, Bansal M, Salama MDM, Ghor SA, Ahmad F (2025) Protective effects of frankincense oil on wound healing: downregulating caspase-3 expression to facilitate the transition from the ınflammatory to proliferative phase. Pharmaceuticals 18(3):407 40143183 10.3390/ph18030407 PMC11945088

[CR83] Wang Z, Bi Y, Li K, Song Z, Pan C, Zhang S, Lan X, Foulkes NS, Zhao H (2023) Nickel oxide nanoparticles induce developmental neurotoxicity in zebrafish by triggering both apoptosis and ferroptosis. Environ Sci: Nano 10(2):640–655

[CR84] Wieland S, Balmes A, Bender J, Kitzinger J, Meyer F, Ramsperger AF, Roeder F, Tengelmann C, Wimmer BH, Laforsch C, Kress H (2022) From properties to toxicity: comparing microplastics to other airborne microparticles. J Hazard Mater 428:128151 35042167 10.1016/j.jhazmat.2021.128151

[CR85] Xu S, Xiaojing L, Xinyue S, Wei C, Honggui L, Shiwen X (2021) Pig lung fibrosis is active in the subacute CdCl2 exposure model and exerts cumulative toxicity through the M1/M2 imbalance. Ecotoxicol Environ Saf 225:112757 34509164 10.1016/j.ecoenv.2021.112757

[CR86] Yalçınkaya Ö, Erdoğan H (2012) Preconcentration and determination of manganese and nickel from various water samples by nano zirconium oxide/boron oxide. Spectrosc Lett 45(8):602–608

[CR87] Yang Z, Tian X, Shu W, Yang Y, Xu J, Kan S (2024) Combined toxicity of polyethylene microplastics and nickel oxide nanoparticle on earthworm (*Eisenia andrei*): oxidative stress responses, bioavailability and joint effect. Environ Sci Pollut Res 31(24):34910–34921 10.1007/s11356-024-33512-6 38713352

[CR88] Yang Y, Sun Y, Gu T, Yan Y, Guo J, Zhang X, Pang H, Chen J (2025) The metabolic characteristics and bioavailability of resveratrol based on metabolic enzymes. Nutr Rev 83(4):749–770 39520710 10.1093/nutrit/nuae161

[CR89] Yang Y, Xiong G, Shi H, Peng Y, Liu J, Jiang Y, Lu M, Liu H, Liu Y (2025) Sinensetin attenuates hepatic ischemia-reperfusion injury through suppressing GRP78/CHOP-mediated endoplasmic reticulum (ER) stress in mice. Front Pharmacol 16:1519497 40012630 10.3389/fphar.2025.1519497 PMC11861360

[CR90] Yildiz Deniz G, Geyikoglu F, Altun S (2024) The regulatory effects of pomiferin dietary on nickel-induced hepatic injury in Sprague-Dawley rats; action mechanisms and signaling pathways. Toxicol Mech Methods 34(5):484–494 38223921 10.1080/15376516.2023.2301667

[CR91] Yuluğ E, Türedi S, Alver A, Türedi S, Kahraman C (2013) Effects of resveratrol on methotrexate‐induced testicular damage in rats. Sci World J 2013(1):489659 10.1155/2013/489659 PMC3745984 23983634

[CR92] Zeng G, Liang J, Jie X, Chen Y, Qi L, Wu Z, Wu S, Li Y (2024) Resveratrol modulates ferroptosis: promising therapeutic targets in ischemia-reperfusion. J Funct Foods 122:106520

[CR93] Zhou S, Li H, Wang H, Wang R, Song W, Li D, Wei C, Guo Y, He X, Deng Y (2023) Nickel nanoparticles induced hepatotoxicity in mice via lipid-metabolism-dysfunction-regulated inflammatory injury. Molecules 28(15):5757 37570729 10.3390/molecules28155757 PMC10421287

